# Atomistic processes of surface-diffusion-induced abnormal softening in nanoscale metallic crystals

**DOI:** 10.1038/s41467-021-25542-2

**Published:** 2021-09-02

**Authors:** Xiang Wang, Sixue Zheng, Shuhei Shinzato, Zhengwu Fang, Yang He, Li Zhong, Chongmin Wang, Shigenobu Ogata, Scott X. Mao

**Affiliations:** 1 Department of Mechanical Engineering and Materials Science, University of Pittsburgh, Pittsburgh, PA USA; 2 Department of Mechanical Science and Bioengineering, Osaka University, Osaka, Japan; 3 Environmental Molecular Sciences Laboratory, Pacific Northwest National Laboratory, Richland, WA USA

**Keywords:** Nanowires, Mechanical properties

## Abstract

Ultrahigh surface-to-volume ratio in nanoscale materials, could dramatically facilitate mass transport, leading to surface-mediated diffusion similar to Coble-type creep in polycrystalline materials. Unfortunately, the Coble creep is just a conceptual model, and the associated physical mechanisms of mass transport have never been revealed at atomic scale. Akin to the ambiguities in Coble creep, atomic surface diffusion in nanoscale crystals remains largely unclear, especially when mediating yielding and plastic flow. Here, by using in situ nanomechanical testing under high-resolution transmission electron microscope, we find that the diffusion-assisted dislocation nucleation induces the transition from a normal to an inverse Hall-Petch-like relation of the strength-size dependence and the surface-creep leads to the abnormal softening in flow stress with the reduction in size of nanoscale silver, contrary to the classical “alternating dislocation starvation” behavior in nanoscale platinum. This work provides insights into the atomic-scale mechanisms of diffusion-mediated deformation in nanoscale materials, and impact on the design for ultrasmall-sized nanomechanical devices.

## Abstract (web summary)

As the sample size goes down to the nanoscale, the surface-related mechanism plays an important role in the deformation of nanoscale crystals. Here, the authors report breakdown of the traditional Hall-Petch-like relation in nanoscale Ag attributed to diffusion-involved nucleation behaviors.

## Introduction

Controlling mechanical properties in the design of new materials through manipulation of microstructural length scale has attracted a great deal of interest in the field of materials science and engineering. As described by Hall–Petch strengthening rule, grain refinement leads to a strength increase in nanocrystalline (NC) metals, due to the reduction of dislocation-nucleation sources^1,2^. As the grain size decreases to a few nanometers (<10 nm), intragrain dislocation activity gives way to GB-mediated mechanisms, including GB sliding^3^, grain rotation^4^, GB migration^5,6^, and diffusional creep^7,8^, which are candidate deformation mechanisms for inverse Hall-Petch relation^9,10^. Analogous to the impact of GBs on the plastic deformation of NC materials, free surface plays an essential role in mediating the plasticity of nanocrystals or nanowires (NWs) with ultrahigh surface-to-volume ratio^11^.

On account of the scarcity of the effective dislocation sources inside NWs, free surface, an effective sink and source of dislocations, governs yielding behavior, leading to the well-known ‘smaller is stronger’ in NWs^12–15^. Besides, akin to the role of GBs in Coble-type diffusion softening behaviors in bulk NC metals, free surface in nanostructured materials can serve as a highway for mass transport^16^. Coble creep presented a conceptual model to describe GB diffusion, but both the concomitant GB sliding and the complex GB structure make the concrete diffusion pathway elusive in polycrystalline materials^7,8^. Contrary to the conjectured diffusion process along grain boundary in classical Coble creep model^7^, the free surface provides a window to visualize the mass transport during creep. Previous studies suggested that as the sample sizes of silver (Ag) crystals decreased below ∼10 nm surface atom diffusion replaced displacive deformation as the dominant deformation mechanism resulting in ‘smaller is much weaker’^17,18^, that, however, have not been corroborated by direct experimental evidence to date. Furthermore, the theory based on the Coble-creep model only gave a plausible description of the softening trend but incapable of providing a consolidated criterion to reveal the inherent transition from a normal to an inverse Hall–Petch regime^16^. Until very recently, Sun et al^19,20^. reported that displacive mechanisms were still activated in the plastic deformation processes of sub-5 nm-diameter Ag NWs. In light of the discrepancy between the fundamentally different plasticity mechanisms activated in the nanostructured metals of comparable size, the validity of the mechanisms proposed in previous studies to account for the so-called inverse Hall–Petch-like effect remains questionable. In addition, Zhong et al.^18^ revealed that slip-activated surface diffusional creep gave rise to a self-healing mechanism, that prevented shear localization and the tendency of plastic instability, leading to super-elongation in Ag NWs. However, whether or not such surface diffusional creep contributed to plasticity and hence caused stress relaxation during plastic flow was not identified in their work. On account of some ambiguity in previous studies, leaps, not strides, need to be taken to bolster our understanding of the atomic-scale mechanism governing strength and plastic flow in small-sized metals, which are of importance not only for drawing a comprehensive mechanistic picture of size-dependent deformation but also for the mechanical reliability and stability of nanoelectronics.

Here, in situ high-resolution transmission electron microscopy (HRTEM) tensile testing technique complemented by molecular dynamics (MD) simulations are performed to investigate the yielding and plastic flow behavior in nanoscale Ag and platinum (Pt). The physical creep processes of the surface mass transport are successfully captured. We reveale that the yield strength-size relationship transits from ‘smaller is stronger’ to ‘smaller is weaker’ with the reduction of sample diameters of Ag crystals, while nanoscale Pt shows traditional Hall-Petch-like-strengthening, i.e., ‘smaller is stronger’. Dislocation nucleation and plastic flow evolution involved by surface creep processes are further analyzed by both direct experimental evidence and complementary molecular dynamic simulation. Based on in situ observation on the physical creep process and mechanical behaviors, the mechanisms proposed in this study catch sights of understanding diffusion-mediated deformation in nanoscale metallic crystals.

## Results

### Strength-size dependences in nanoscale Ag and Pt

**Fig. 1:**
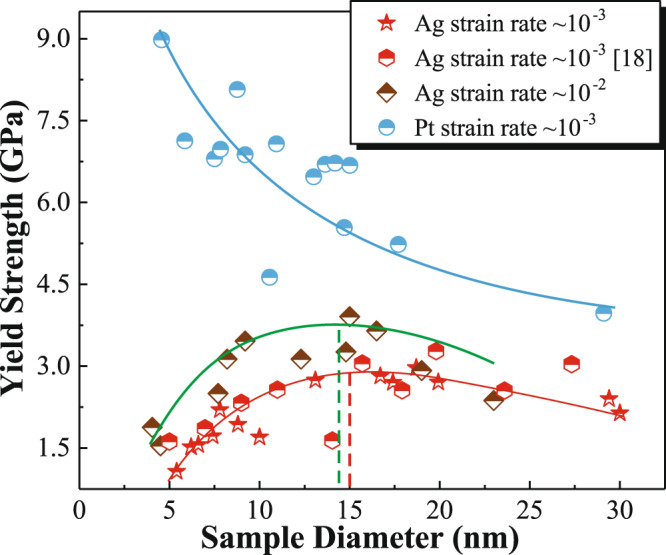
Size dependence of yield strength in Ag NWs and Pt NWs. In situ tensile straining of < 112 > -oriented Ag NWs with different sample diameters were performed at strain rates of ~ 10^−2^ s^−1^ (green points) and ~ 10^−3^ s^−1^ (red points). Pt NWs were loaded along <112 > -orientation at a strain rate of ~ 10^−3^ (blue points). The blue, red and green lines represent the tendency of the strength-size relationship in Pt NWs under a strain rate of ~ 10^−3^ s^−1^ and Ag NWs under a strain rate of ~ 10^−2^ s^−1^ and ~ 10^−3^ s^−1^, respectively.

In situ HRTEM tensile tests were performed along < 112 > -orientation at room temperature under a strain rate of ∼ 10^−3^ s^−1^ to investigate yield strength-size dependence in Ag and Pt NWs (see Methods for details). The yield strength here is defined as the maximum elastic lattice stress that a defect-free crystal can withstand just before the first event of dislocation nucleation. (Supplementary Discussion 1 and Supplementary Figs. 1 and 2). As shown in Fig. 1, the yield strength of Pt NWs increased with decreasing sample size following the classical Hall-Petch-like relationship, that is ‘smaller is stronger’, as previously reported in nanowhiskers^21^ and nanopillars^22^. In contrast, the yield strength-size relationship in Ag NWs changed from ‘smaller is stronger’ to ‘smaller is weaker’ as the sample diameter of Ag NWs decreased below a critical size of ~ 15 nm (the red line in Fig. 1). Given that the strength of NWs is controlled by dislocation nucleation^11,23,24^, these two distinct size effects are speculated to be associated with different nucleation behaviors.

**Fig. 2:**
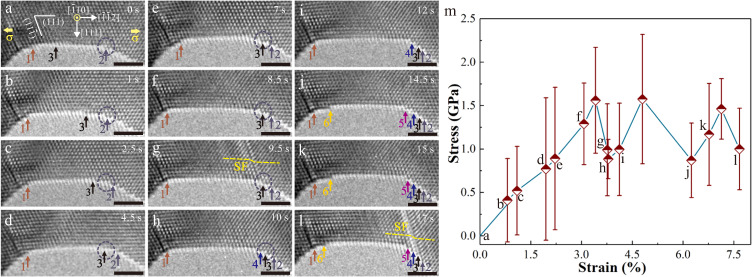
Dislocation nucleation assisted by surface diffusion in an Ag NW. **a**, TEM image of a pristine 6.6-nm-diameter Ag NW under < 112 > tensile loading at room temperature and a strain rate of ~ 10^−3^ s^−1^. **b**–**f**, Sequential TEM images showing the lateral migration of three atomic steps (surface atom diffusion) on the {111} surface of the Ag NW. The initial position of step 2 was marked by a navy blue circle. **g**, A partial dislocation activity observed at the overlapping position of steps 2 and 3. **h**, A one-layer surface step (step 4) formed by the elimination of a stacking fault. **i-l**, Sequential TEM images showing the plastic flow of the Ag NW with a small number of surface diffusion. All scale bars are 2 nm. Each surface step is tracked by an arrow of specific color. **m**, Lattice stress versus applied strain curve during tensile loading; points (**a**–**l**) indicate the states of deformation shown in the TEM images of **a**–**l**. The error bars represent the variations of the measured lattice stresses at different locations of the nanowire.

**Fig. 3:**
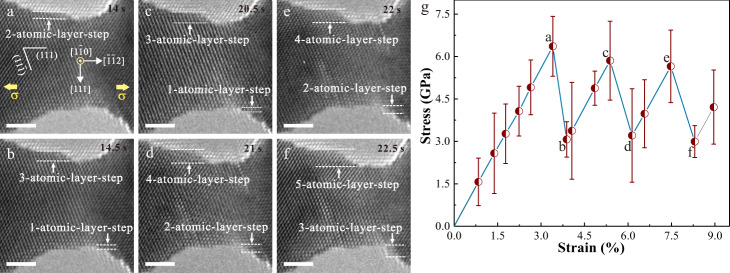
In situ TEM tensile testing shows pure displacive deformation in a Pt NW. **a-f** A series of TEM images showing the plastic deformation process of a 5.9-nm-diameter under tensile loading along < 112 > -orientation at room temperature under a strain rate of ~ 10^−3^ s^−1^. The plasticity of Pt NW is controlled by repeated nucleation and emission of full dislocations, and each full dislocation activity resulted in the surface steps thickened by one atomic layer (**b**, **d**, **f**). Obvious surface atom diffusion was not observed during tensile loading causing that surface steps remained immobile. All scale bars are 2 nm. **g** Lattice stress versus applied strain curve during tensile loading; points (**a**–**f**) indicate the states of deformation shown in the TEM images of **a–f**. The error bars represent the variations of the measured lattice stresses at different locations of the nanowire.

To uncover the underlying mechanisms governing different nucleation behaviors, atomic-scale deformation processes of Ag and Pt NWs were systematically investigated, as shown in Figs. 2 and 3 and Supplementary Movies 1 and 2. Some blurry boundaries in the HRTEM images were attributed to the delocalization effect instead of surface contamination^25^. After fabrication, three surface defects (surface atomic steps) existed in the 6.6-nm-diameter Ag NW (Fig. 2a). The lateral movements of surface atomic steps, arose from curvature- and stress-driven surface atom diffusion^17–19^, occurred before surface dislocation nucleation. As shown in Fig. 2b–d, steps 2 and 3 migrated to the right side, while step 1 migrated to the left side upon tensile loading (See Supplementary Movie 1 for more details of the step migration process). As step 3 migrated to the initial location of step 2 (indicated by a navy blue circle), a partial dislocation nucleated and propagated from this site, leaving behind a stacking fault (SF), as shown in Fig. 2e–g. Subsequently, the SF was eliminated by a trailing partial at the same site, leaving behind a one-atomic-layer step at surface (step 4)^11,26^, as shown in Fig. 2h. Instead, the eventual site of moving step 1 did not favor dislocation nucleation, which was associated with the different surface diffusional processes. Surface atom diffusion via the overlap of surface defects (steps 2 and 3) changed surface atomic configuration and thus lowered nucleation stress, that is, yield strength^27,28^. In contrast to Ag NWs, the migration of surface steps, namely, surface diffusion was not observed before dislocation nucleation in Pt NWs (Fig. 3 and Supplementary Movie 2). This difference was attributed to the high activation energy barrier and low surface mobility for self-diffusion in Pt, compared to Ag (Supplementary Table 1)^18,29^.

In addition to the direct experimental observations, activation volume $({\Omega })$, that is an effective kinetic signature of deformation mechanism^12,30–32^, as well as activation free energy $(\varDelta G)$ were determined to confirm the operation of surface-diffusion-assisted nucleation mechanism in Ag NWs (Supplementary Discussion 2). The calculated ${\Omega }$ and $\varDelta G$ for Ag NWs (<15 nm) are $\sim 0.24\,{b}^{3}$ and $\sim 0.063\,{{{{{\rm{eV}}}}}}$, respectively, where *b* is the Burgers vector for a perfect dislocation. These values are of the same order as the theoretical values ($\sim 0.1\,{b}^{3}$^12^ and $0.044{-}0.3\,{{{{{\rm{eV}}}}}}$^29,33,34^) for surface diffusion in Ag. In contrast, the calculated ${\Omega }$ and $\varDelta G$ for the Pt NWs ($\sim 1.02\,{b}^{3}$ and 0.316 eV) and the Ag NWs with diameters above 15 nm ($\sim 1\,{b}^{3}$ and 0.141 eV), are consistent with the theoretically predicted values for surface dislocation nucleation without diffusional events, where ${\Omega }$ and $\varDelta G$ are in the range of $1{-}10\,{b}^{3}$ and 0.16–0.34 eV^35,36^, respectively. Thus, it is plausible to conclude that the diffusional mechanism facilitates dislocation nucleation in Ag NWs (<15 nm), whereas, conversely, dislocation nucleation in Ag NWs (>15 nm) (Supplementary Fig. 4) and Pt NWs was not associated with diffusional deformation.

Considering that the diffusional deformation is sensitive to the strain rate^7,27,31^, to further probe the surface-diffusion-assisted nucleation, which is a rate-controlling process the yield strength-size dependence of Ag NWs at a higher strain rate of ~ 10^−2^ s^−1^ was investigated. As shown in Fig. 1, the yield strength of Ag NWs increased with the applied strain rate, and the critical size for softening was smaller, which may be attributed to the less diffusional events prior to dislocation nucleation at a higher strain rate. Several lines of direct observational evidence and theoretical calculations mentioned above indicated that two distinct nucleation mechanisms were operative in Ag NWs, that is, surface dislocation nucleation and surface-diffusion-assisted dislocation nucleation. Analogous to GB in NC materials, free surface in NWs plays an increasingly important role in mediating plasticity with the reduction of sample size^32,37^. Surface dislocation nucleation and subsequent dislocation starvation governed the plasticity of nanoscale crystals^14,15,35,38^. Based on surface-dislocation-nucleation-theory^14,35^, the effective nucleation sites were reduced with decreasing sample size, resulting in the “smaller is stronger” observation in Pt NWs and Ag NWs (>15 nm). Free surface, having reduced constraints and larger diffusivity, can also act as a highway for mass transport in NWs^16^. Since activation energy for atomic diffusion on a low-indexed surface is small, surface atom diffusion can be activated in the nanometer regime and becomes stronger with the reduction of sample size^17–19,39^. Consequently, surface diffusion served as a precursor mechanism for dislocation nucleation resulting in a “smaller is weaker” trend in Ag NWs (<15 nm).

### Atomic-scale mechanisms of surface-diffusion-assisted dislocation nucleation

**Fig. 4:**
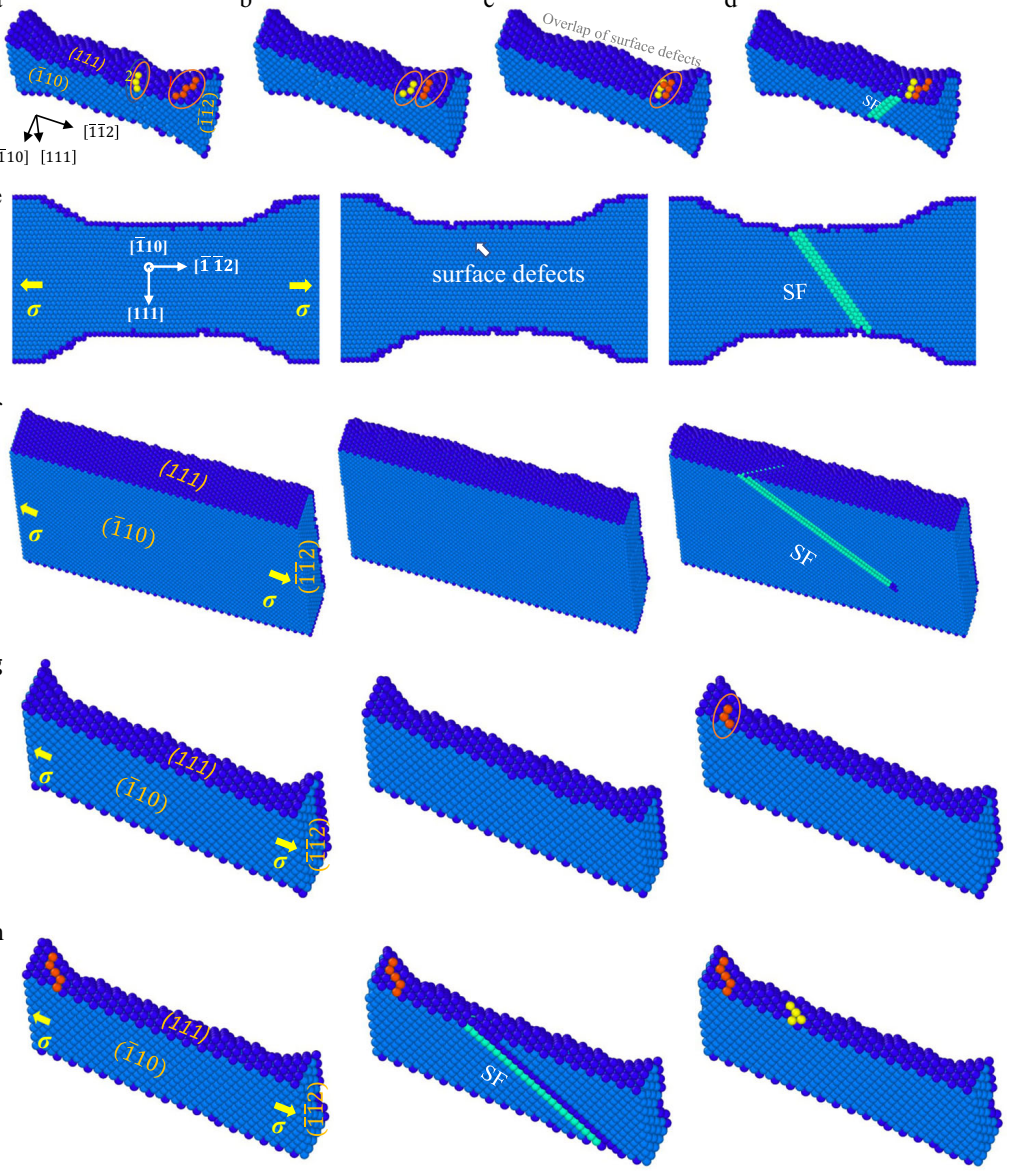
MD simulations of dislocation nucleation mechanism in metallic NWs under < 112 > tension at the temperature of 800 K and a strain rate of 10^5^ s^−1^. **a** Two atomic steps (steps 1 and 2) formed by surface diffusion in a 6-nm-diameter Ag NW. **b** Surface-diffusion-assisted migration of atomic steps (steps 1 and 2) to the right side of NW. **c** Overlap of surface defects. **d** Nucleation and propagation of leading partial dislocations at the overlapping position of surface defects. **e** Dislocation nucleation assisted by single-atom diffusion at the surface of a 6-nm-diameter Ag NW in a random and chaotic way. **f** Dislocation nucleation in a 20-nm-diameter Ag NW with weak surface diffusion. **g** Dislocation nucleation in a 6-nm-diameter Pt NW with nearly no surface diffusion. **h** No diffusional events during plastic flow in the Pt NW causing that surface steps remained immobile. Surface steps caused by full dislocation activities are colored by orange and yellow in **g**, **h**. The color coding corresponds to surface atoms in blue color, atoms in SFs in light green color, and atoms in perfect face-centered cubic (FCC) crystal arrangement in light blue.

Albeit the inherent high strain rate in MD simulations make it difficult to compare deformation mechanisms in experiments and simulations, the temperature of 800 K and the strain rates of 10^5^ s^−1^ and 10^7^ s^−1^ were chosen in our simulations such that surface diffusion, if any, only occurred near free surface (Supplementary Figs. 5, 6, 8 and 9; see Methods for details), which is a salient characteristic of surface diffusion in nanocrystals at room temperature^17–19,26,40^. As shown in Fig. 4a, Supplementary Figs. 7a, b and Supplementary Movie 3, two one-atomic-layer steps (steps 1 and 2), formed by surface diffusion, appeared at the surface of a 6-nm-diameter Ag NW. With further loading, the moving step 2 overlapped step 1 forming a two-atomic-layer step, and then partial dislocations nucleated from the newly formed step (Fig. 4b–d and Supplementary Figs. 7c-f), similar to the experimental observations (Fig. 2a–g). The increase in step height via the overlap of surface defects increased local stress concentration near the overlapping position^26,41,42^, and thus lower nucleation stress, favoring dislocation nucleation from this site. Additionally, the computational results of 6-nm-diameter Ag NWs also showed that localized diffusion of individual atoms in a random and chaotic way turned initially smooth surfaces into potholed surfaces, creating some mass-deficient defects, that significantly lower nucleation stress^27,41^ (Fig. 4e and Supplementary Movie 4). It is energetically more favorable to nucleate a dislocation from the mass-deficient defects (Fig. 4e). Here, our Ag simulations provide direct computational evidence that long-range and localized atomic diffusion at surface can lower nucleation stress and further induce the inverse Hall-Petch effect. In contrast, weak and nearly no surface diffusion occurred before dislocation nucleation in 20-nm-diameter Ag NW (Fig. 4f and Supplementary Movie 5) and 6-nm-diameter Pt NW (Fig. 4g and Supplementary Movie 6), respectively, in agreement with the experimental evidence that surface diffusion in NWs was size- and material-dependent (Figs. 1 and 3, Supplementary Fig. 4). In these nanoscale metals without surface diffusion, dislocations prefer to nucleate at the middle segment or the corners of the sample with higher stress compared to the other parts of sample^43^, as shown in Fig. 4f–g. With the decrease in the size of the sample without preliminary surface diffusion, the effective nucleation sites were reduced, resulting in “smaller is stronger”^14,35^. For all the Ag and Pt NWs, dislocation always nucleated in the gauge section, and surface diffusion, if any, remained confined to free surface during tensile loading (Supplementary Fig. 8). Besides, the yielding of 6-nm-diameter and 20-nm-diameter Ag NWs at a higher strain rate of 10^7^ s^−1^ were also shown to be controlled by dislocation nucleation with and without preliminary diffusion events, respectively (Supplementary Fig. 9), consistent with the size effect on yielding observed experimentally (Fig. 1). Compared with surface dislocation nucleation, the surface-diffusion-assisted nucleation mechanism led to obviously increased strain-rate sensitivity of nucleation stress owing to its small activation volume (Supplementary Fig. 10).

### Surface-atom-diffusion-induced softening during plastic flow

**Fig. 5:**
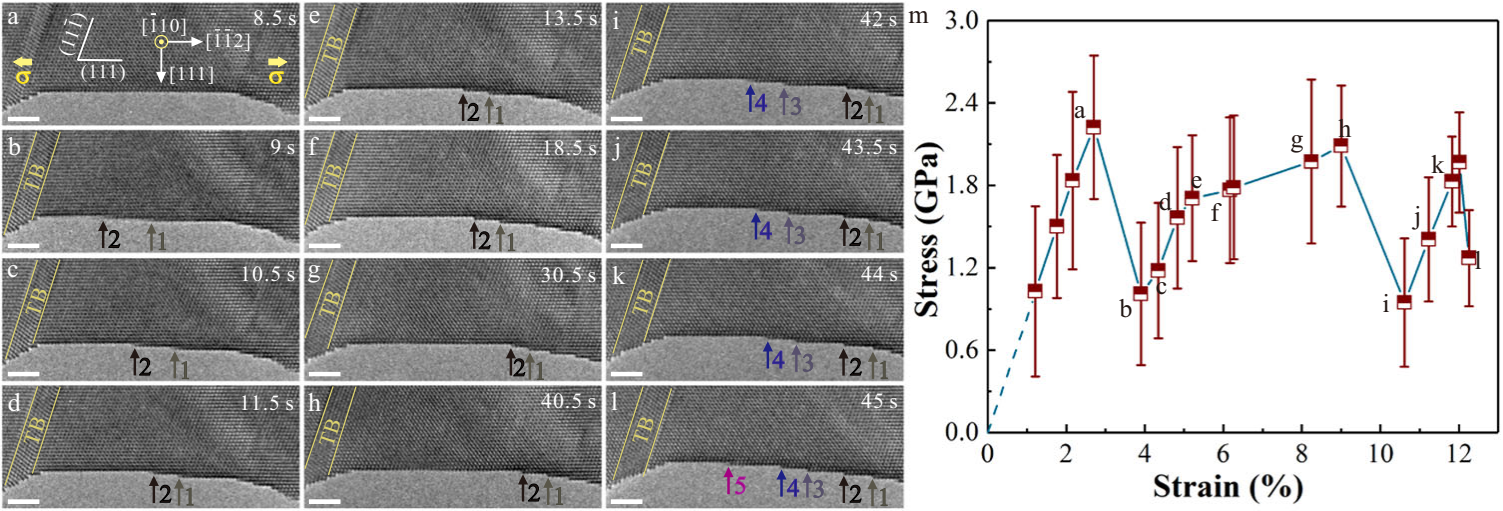
Surface atom diffusion induced abnormal softening during plastic flow in an Ag NW. **a** Pristine Ag NW with a diameter of 13.1 nm as viewed along $[\bar{1}10]$ and loaded along $[\bar{1}\bar{1}2]$-orientation at room temperature and a strain rate of ~ 10^−3^ s^−1^. **b** Two surface steps (steps 1 and 2) formed by full dislocation activities. **c-h**, Surface-diffusion-assisted migration of surface steps (steps 1 and 2) to the right side of the NW during tensile loading. **i** Two new surface steps (steps 3 and 4) in NW formed by full dislocation activities. **j-l**, Sequential TEM images showing the plastic flow of the Ag NW with a small number of surface diffusion. All scale bars are 2 nm. Each surface step is tracked by an arrow of a specific color. **m** Lattice stress versus applied strain curve during tensile loading corresponding to the sequence in **a–l**. The initial strains were nominalized by subtracting the plastic strain in the previous plastic deformation history. The error bars represent the variations of the measured lattice stresses at different locations of the nanowire.

To reveal the effect of surface diffusion on plastic flow, the lattice stress-applied strain relations and the corresponding mechanical behaviors in Ag NWs were investigated (Figs. 2 and 5 and Supplementary Fig. 11, and Supplementary Movies 1, 7 and 8). The applied strains were determined from the length variation of the gauge section which is chosen based on the geometry of samples (Supplementary Fig. 12). At the early stage of tensile deformation without detectable diffusional events, the lattice stress increased with the applied strain linearly (before point a in Fig. 5m and Supplementary Fig. 11i). As the lattice stress increased to a yield point, plastic deformation occurred with dislocation nucleation, subsequent propagation across the NW and eventual annihilation at free surface. Such displacive deformation partly released elastic lattice stress inside NW and thus led to very precipitous stress drops (Fig. 2g, h, j, l, Fig. 5b, i, l and Supplementary Figs. 11b, f, h), as reported previously^19,44^. The differences between the experimental and theoretical values of strain increments, that correspond to different stress drops, were generally less than 0.05% (Supplementary Discussion 3 and Supplementary Table 3), verifying that the evaluation method for the stress-strain curves in this work is feasible and reliable. After the yielding of 13.1-nm-diameter Ag NW, two atomic steps, induced by full dislocation activities^11,18,26^, formed at the surface (Fig. 5b), and then continuously migrated to the right edge with further loading (Fig. 5c-h), driven by the chemical potential difference between the middle thin regions and terminal thick regions^18,19^. Meanwhile, the flow stress-strain behavior when surface diffusional creep happened cannot be described well by a linear elastic relationship before the next plastic event (points b-h in Fig. 5m), which was also observed in 30-nm-diameter Ag NW (points b-e in Supplementary Fig. 11i). In contrast, no obvious softening occurred during plastic flow with very limited surface diffusion (points i-k in Fig. 5m, points f-g in Supplementary Fig. 11i and points h-l in Fig. 2m). The details of surface diffusion and dislocation activities in Ag NWs are depicted in Supplementary Discussion 4.

Different from the alternatively elastic rise and drop of the lattice stress in most NWs^37,44^, such an unusual softening behavior during plastic flow in Ag NWs is associated with surface diffusional creep. When slip-activated surface diffusional creep occurs, the increasing rate of flow stress $\dot{\sigma }$ at any given applied strain can be expressed as

1 $$\dot{\sigma }=\frac{E}{100}\cdot \frac{\dot{\varepsilon }-{\dot{\varepsilon }}_{{{{{{\rm{diffusion}}}}}}}}{\dot{\varepsilon }},$$

where $\dot{\varepsilon }$ is the applied strain rate, and ${\dot{\varepsilon }}_{{{{{{\rm{diffusion}}}}}}}$is the plastic strain rate accommodated by surface diffusion, which increases continually with lattice stress/strain^39^. Hence, ${\dot{\varepsilon }}_{{{{{{\rm{diffusion}}}}}}}$ progressively reduced the increasing rate of stress versus strain during tensile loading, in accordance with experimental evidence. The observed diffusional deformation could partly contribute to the plastic strain of NWs. (Supplementary Fig. 13) Our MD simulations also demonstrated that surface atoms could diffuse into the interior of NW, contributing to plastic strain (Supplementary Figs. 15 and 16). In contrast to the experimental results, the stress-strain curve before yielding was linear in MD (Supplementary Fig. 17), despite a wealth of atomic diffusion occurred at surface. Such difference may be attributed to the fact that the strain rate in MD simulations (10^5^ s^−1^) is 8 orders of magnitude higher than that in our experiments (~ 10^−3^ s^−1^). According to our experimental results, ${\dot{\varepsilon }}_{{{{{{\rm{diffusion}}}}}}}$ was of the same order as the experimental strain rate, and thus was too small to influence the shape of the stress-strain curve in MD. Different from the coupled displacive-diffusive deformation process in Ag NWs, the migration of surface steps, namely, no obvious surface diffusion was observed during plastic flow in Pt NWs (Fig. 3), which was also captured in MD simulations (Fig. 4g, h). The plasticity in Pt NWs was mediated by a pure displacive mechanism where each full dislocation activity increased the step height by one atomic layer (Fig. 3b, d, f)^11,18,26^. Consequently, the stress-strain curve in Pt NW before dislocation nucleation (points a, c, and e in Fig. 3g) was described well by linear elastic relationship, consistent with alternating dislocation starvation behavior in NWs^37,44^. Surface diffusional creep, accommodating plastic strain, effectively delayed the progressive activation of the following dislocations and thus weakened the hardening effect resulted from dislocation starvation^11,12,24,37,44^, leading to an abnormal softening during plastic flow. In addition to softening, surface creep, overwhelmingly dominating over displacive deformation, may raise the probability of stress concentration and premature necking, instead of superelongation benefiting from the compatible interplay of displacive and diffusive mechanism^18^.

## Discussion

The yield strength of nanoscale crystals, controlled by surface dislocation nucleation, can be expressed as^35^

2 $${\sigma }={\hat{{\sigma }}}_{{{{{{\rm{ath}}}}}}}-\frac{{{{{{{\rm{k}}}}}}}_{{{{{{\rm{B}}}}}}}T}{{\Omega }}\,{{{{{\mathrm{ln}}}}}}\left(\frac{{{{{{{\rm{k}}}}}}}_{{{{{{\rm{B}}}}}}}{T}{\tilde{N}}{v}_{\rm D}}{E\dot{\varepsilon }{\Omega }}\right),$$

where k_B_ is the Boltzmann constant, ${\upsilon }_{{{{{\rm{D}}}}}}$ is the attempt frequency, ${\hat{{\sigma }}}_{{{{{{\rm{ath}}}}}}}$ is athermal stress and $\tilde{N}$ is the number of dislocation-nucleation sites. For the Pt and Ag NWs (>15 nm), nucleation sites $(\tilde{N})$ decreased with size causing Hall-Petch-like-strengthening^13,14,35^. Notably, the incipient plasticity, that is dislocation nucleation, was supposed to occur at sites where the relative interatomic displacements exceed the critical value^14,15^. Especially for Pt NWs with negligible diffusional activities, surface diffusion cannot create surface defects, favoring dislocation nucleation. The effective nucleation sites for dislocation in Pt NWs resulted from the initial defect sites, which decrease with reducing sample size^14,15^, leading to the “smaller is stronger” trend. For the Ag NWs (<15 nm), coupled displacive-diffusive mechanism leads to a ‘smaller is weaker’ trend, which is attributed to the following reasons. First, with decreasing size, diffusion of the individual atom becomes stronger^17–19,39^, and introduce more surface defects (Fig. 4e), leading to an increase in the nucleation sites. The driving force for the surface diffusion and atom diffusivity would increase with the reduction of the sample size^16,45^. (Supplementary Equations. 8–11) Second, the activation volume is reduced due to a mechanism transition from displacive deformation (1–10 b^3^) to diffusional deformation (~ 0.1 b^3^)^12^. Third, the overlap of surface defects changed surface configuration (Fig. 2e, f and Fig. 4c), reducing the athermal nucleation stress $({\hat{\sigma }}_{{{{{{\rm{ath}}}}}}})$ directly^27,28,42,46^. (Supplementary Equation. 12) Considering all the factors mentioned above, the yield strength of Ag NWs (<15 nm), based on Eq. (1), decreases with size, causing the breakdown of the Hall–Petch-like relation. (See Supplementary Discussion 5) The critical size scale for the breakdown of Hall-Petch-type relation will decrease and even disappear with increasing applied strain rate or decreasing experimental temperature, since less diffusional events occur before dislocation nucleation^16,47^. Surface-diffusion-assisted nucleation mechanism should be highly sensitive to some intrinsic properties, e.g. sample size^17,18,40^, surface configuration^13,27^, activation energy barrier for atomic diffusion^29,48^, as well as some extrinsic properties, e.g. loading conditions, strain rate, experimental temperature^16,49^. Given the complexity and uncertainty of diffusional deformation^12^, the complex interplay among these factors should await systematic future research.

Previous studies suggested that as the size of Ag nanoparticles decreased below ∼10 nm, plastic deformation should be controlled by surface atom diffusion without dislocation activity, which was expected to cause inverse Hall–Petch-like relation^17,18^. In contrast, our work painted a continuous picture of the size dependence of yield strength, and directly revealed that aside from pure diffusive mechanism, surface-diffusion-assisted dislocation nucleation, namely, coupled displacive-diffusive mechanism, truncated the ‘smaller is stronger’ trend and turned it into ‘smaller is weaker’. A recent experimental study on pristine Pd NWs also demonstrated that coupled diffusive-displacive processes could govern plastic deformation instead of pure crystal slip or diffusional creep, causing that ‘smaller is stronger’ no longer holds^30^. The previous research showed that the interplay between compressive loading and tensile surface stress in nanocrystals could also cause ‘smaller is weaker’^13^. However, such loading-dependent properties did not reflect the intrinsic effect of size scale^13^. Besides, the initial interior stress in sample influenced the critical dislocation-nucleation stress^13^. In our study, the pristine nanocrystals or NWs generated by Joule-heating welding^23^ weakens the interior stress effect from the defect debris. In addition to the scenario of softening, the surface oxidation and triaxial loading could suppress the diffusion and surface dislocation-nucleation and may lead to the size-independent strength plateau^50^. Given that free surfaces in nanocrystals have some similar features as GBs in NC metals when mediating displacive and diffusive deformation, small-volume single crystals can be regarded as isolated small grains in NC metals roughly. The coupled displacive-diffusive mechanism studied here may provide atomic-scale insights into interplays between intragranular crystal slip and GB-mediated diffusive processes in bulk NC metals, especially in metals with relatively low melting temperatures.

Coble creep is a classical model for describing the diffusional transport of atoms along GBs, which quantitatively interprets the plastic strain accommodated by diffusive processes in nanocrystalline metals at room temperature^7^. Different from the driving force for Coble creep resulted from vacancy concentration gradient with applied stress^7^, the difference in chemical potential caused by curvature and stress drives atomic diffusion along free surface in nanoscale metals (see details in Supplementary Discussion 5)^16–18,47^. The atoms at a surface step with high chemical potential tend to migrate towards the end of the NW with low chemical potential (Supplementary Fig. 14), following the pathway for surface atomic diffusion proposed by Zhong et al.^18^. The atoms at the step edge on {111} planes sequentially hop along < 112 > direction to climb the surface step^48^, leading to the experimental observation of the continuous lateral movement of the surface steps (Figs. 2 and 5). Assuming the diameter of the Ag NW is ~ 10 nm, the flux per second for surface step motion with an average velocity of 0.2–1 nm s^−1^ was 0.4–1 × 10^−17^ m^2^ s^−1^, which was comparable in magnitude with the curvature-driven surface diffusivity in Ag nanoparticles (~ 5 × 10^−17^ m^2^ s^−1^)^16,18^. Though our TEM observation fell short in capturing the localized random diffusion of individual atoms at surface directly, its diffusional pathway was supposed to be similar to that for surface step migration^29^. Considering that the direct observation of the process of mass flow along GBs is still lack, our work gives insights into understanding the diffusional processes of the surface-assisted Coble-type creep^17^ and the coupled diffusive–displacive deformation mechanism in nanoscale metals.

In conclusion, the combined in situ HRTEM tensile tests and MD simulations have presented the surface-mediated mass transport in nanoscale Ag, and revealed that the yield strength-size relationship changed from ‘smaller is stronger’ to ‘smaller is weaker’ with decreasing the sizes of Ag crystals, whereas nanoscale Pt showed traditional “Hall-Petch-like” behavior. Both direct experimental and computational results demonstrated that surface-diffusion-assisted dislocation nucleation was responsible for the inverse Hall-Petch-like effect. Our work also unveiled that surface atom diffusion significantly lowered flow stress during mechanical loading in nanoscale Ag, which was quite different from the pure displacive plasticity in nanoscale Pt. Broadly, our work visualized the physical surface creep processes at atomic-scale and developed an integrated model to reveal the strength-size dependence of nanoscale materials, which sheds light on the atomistic mechanism of surface diffusion-involved displacive deformation in nanoscale materials.

## Methods

**In situ tensile testing.** In situ tensile tests of Ag NWs and Pt NWs were performed inside a FEI Tecnai F30 TEM and a FEI Titian 80-300 TEM using a Nanofactory scanning tunneling microscope (STM) holder. The metals investigated in this work are high-purity (99.999%) Ag and Pt provided by ESPI Metals. Before the in situ tensile tests, the Ag and Pt nano-tips were generated at the clean fracture surface of bulk metallic substrates using a wire cutter and then loaded onto the static side of the TEM holder. The approach in this work produced nano-tips with clean surfaces, which were ideal for studying surface diffusive plasticity in Ag NWs. Plasma cleaning was conducted on the Ag and Pt nanotips before all the in situ TEM tests to reduce the potential effects of carbon deposition on surface diffusion. The sharp nano-tips of Ag and Pt oriented in the < 110 > zone axis were selected to be welded together with the probe side by applying a constant voltage of ~ 1 V^18^. When the nano-tip contacted with the probe side, the pre-applied potential could melt the nano-tip and then generated bridge-shaped single-crystal nanowires with controllable dimensions, which were epitaxially grown from one substrate and connected to either a tungsten probe or another substrate at the opposite side. The cross-section of the as-fabricated NW is nearly circular with equal width and thickness^23^. The middle segment of the single-crystalline nano-bridge had a smaller diameter compared to those at either end, serving as a gauge section. The strain rate of ~ 10^−2^ s^−1^ and ~ 10^−3^ s^−1^ during tensile loading were controlled by the movement speed of the piezo-manipulator of the STM holder. Besides, all the in situ experiments were recorded in real-time by a charge-coupled device (CCD) camera at a rate of 0.5 s/frame.

All the in situ tensile experiments were operated at 300 kV, and low dose (low electron beam intensity < 10^5^A m^−2^) conditions were used to reduce the influence of electron beam irradiation on surface diffusion in Ag NW^17,18^. Given that the activation energy for surface diffusion is much lower than the sublimation energy in Ag^29,33,34,51^, the rate of curvature- and stress-driven surface diffusion is much higher than the sputtering rate caused by electron beam. Hence, surface atom diffusion can be influenced by electron beam irradiation, but electron-beam effect in our work is not a primary factor. Based on the estimation of the temperature rise induced by the electron beam for Ag nanoparticles in ref. ^17^, the temperature rises in Ag nanoparticles were less than 0.1 k. Due to the short time of tests (less than 3 min), no obvious carbon deposition occurred on the Ag and Pt NWs during tests.

### Molecular dynamics simulations

MD simulations were performed using the Large-scale Atomic/Molecular Massively Parallel Simulator (LAMMPS) package^52^. The embedded atom method (EAM) potentials for Ag developed by Wu and Trinkle^53^ and for Pt by Sheng et al.^54^ were used for interatomic interaction. To investigate the surface atomic diffusion on {111} facets and the related dislocation nucleation events as observed in our experiments, the cross-sectional shapes of the samples in MD simulations were set to be rectangular. Given that surface diffusion is a rate-limiting process^27^, the sample geometry could not change significantly during the observation period (Supplementary Fig. 16). Thin-film samples oriented along $[\bar{1}\bar{1}2]$, $[111]$ and $[1\bar{1}0]$ directions with two different sizes were modeled. The $[1\bar{1}0]$ and $[\bar{1}\bar{1}2]$ directions were the viewing and loading direction in the experiments, respectively. The supercell size and gauge section length along $[\bar{1}\bar{1}2]$ direction were 25 nm and 9 nm, respectively. The cross-section areas of gauge section were $6\times 2$ and $20\times 6.7$ nm^2^, respectively. A periodic boundary condition was applied along the loading direction. The energy of the models was minimized by the conjugate gradient method, and then relaxed for 100 ps under zero pressure using an NPT ensemble with a Nose-Hoover thermostat at 300, 500, and 800 K, respectively. Following relaxation, tensile deformation at the same temperature was carried out by stretching the simulation box along the $[\bar{1}\bar{1}2]$ direction at a constant engineering strain rate in the NVT ensemble. Two different strain rates of 10^5^ and 10^7^ s^−1^ were studied. The virial stress of the gauge section was calculated as the stress along the loading direction using the undeformed cross-section area at the gauge section. Atomic structures of nanofilm during deformation was studied in the OVITO^55^ using common neighbor analysis (CNA) for the defect structure and the non-affine squared displacements $({D}_{\min} ^{2})$^56^ calculation for diffusivity of atoms. In the CNA study, the average atomic positions of 2 ps MD run for each snapshot were used to reduce the noise due to thermal fluctuation. Notably, based on Wuffle construction theory, the equilibrium Ag crystals consist of {111} and {100} facets^57^. Beyond the interested period investigated in our MD simulation (shown in Fig. 4, Supplementary Figs. 7–9), the cross-sectional shape of the samples changed from the initial rectangle to the polygon, and finally to the nearly-circular shape (hexagonal shape consisted of {111} and {100} planes), as shown in Supplementary Fig. 18.

## Supplementary information

Supplementary Information

Description of Additional Supplementary Files

Supplementary Movie 1

Supplementary Movie 2

Supplementary Movie 3

Supplementary Movie 4

Supplementary Movie 5

Supplementary Movie 6

Supplementary Movie 7

Supplementary Movie 8

## Data Availability

All data needed to evaluate the findings in the paper are present in the paper and/or the Supplementary Information. Additional data related to this paper may be requested from the corresponding author S. X. Mao.
