# Supplementary material for: Atomistic processes of surface-diffusion-induced abnormal softening in nanoscale metallic crystals
Source: Nat Commun. 2021 Sep 2;12:5237. doi: 10.1038/s41467-021-25542-2 (PMC8413310; doi:10.1038/s41467-021-25542-2)
Supplement: Supplementary file 1 — Supplementary Information [file 41467_2021_25542_MOESM1_ESM.pdf]

Supplementary Information

for

Atomistic observation on surface diffusion-controlled softening in metallic nanocrystals

Wang et al.

Supplementary Information includes:

Supplementary Figures 1-18

Supplementary Tables 1-3

Supplementary Discussion

Supplementary References

Supplementary Figures

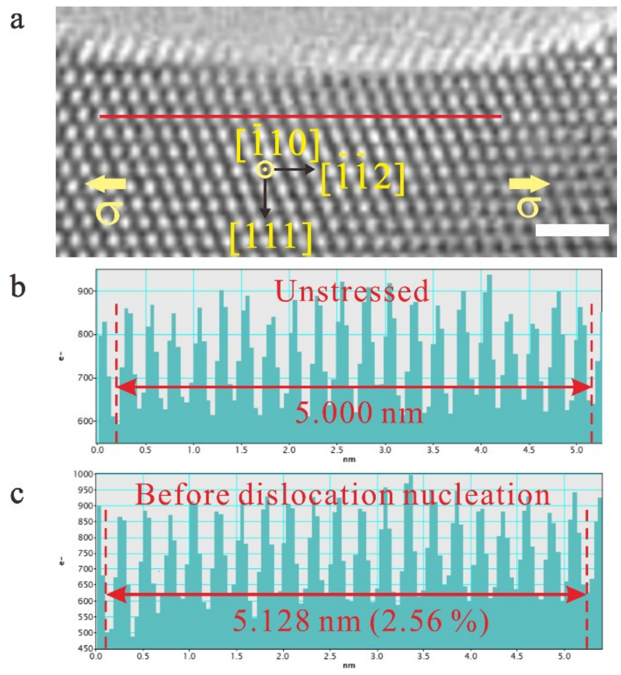

Supplementary Figure 1. Lattice strain measurement of Ag NW under <112> tensile deformation. a, Lattice strain was obtained by measuring lattice expansion along loading direction. The scale bar is 1 nm. **b**, Interplanar spacing over 20 atomic planes of the unstressed state. **c**, Interplanar spacing over 20 atomic planes of the deformed state. An elastic lattice strain of 2.56 % was obtained via comparing the difference in interplanar spacings.

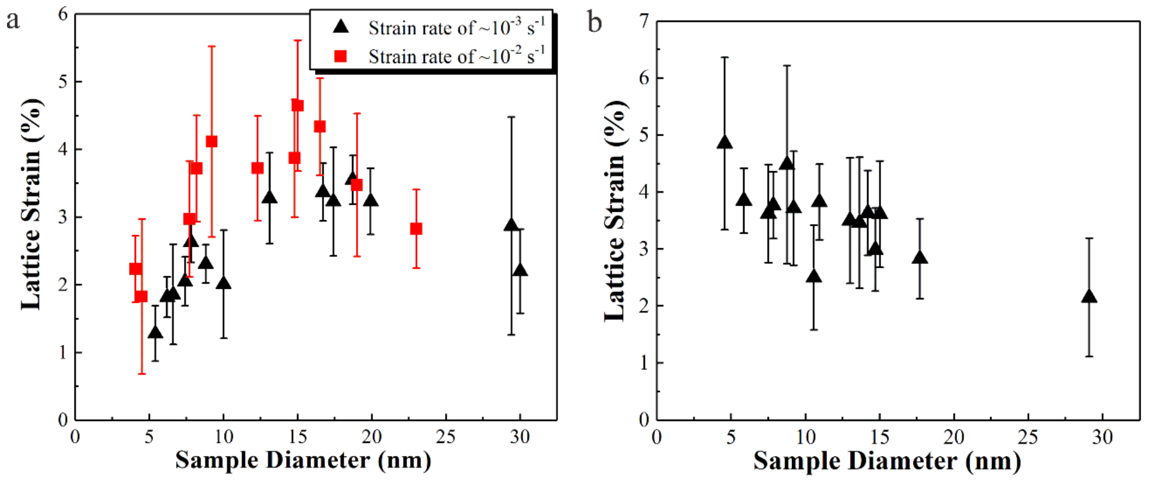

Supplementary Figure 2. Elastic strain limit before yielding in tensile tests of metallic NWs. a, Ag NWs at a strain rate of ~10^{-2} s^{-1} and ~10^{-3} s^{-1}. **b**, Pt NWs at a strain rate of ~10^{-3} s^{-}

[1]. Error bars represent the variation in the lattice strain limits measured at different locations of a nanocrystal.

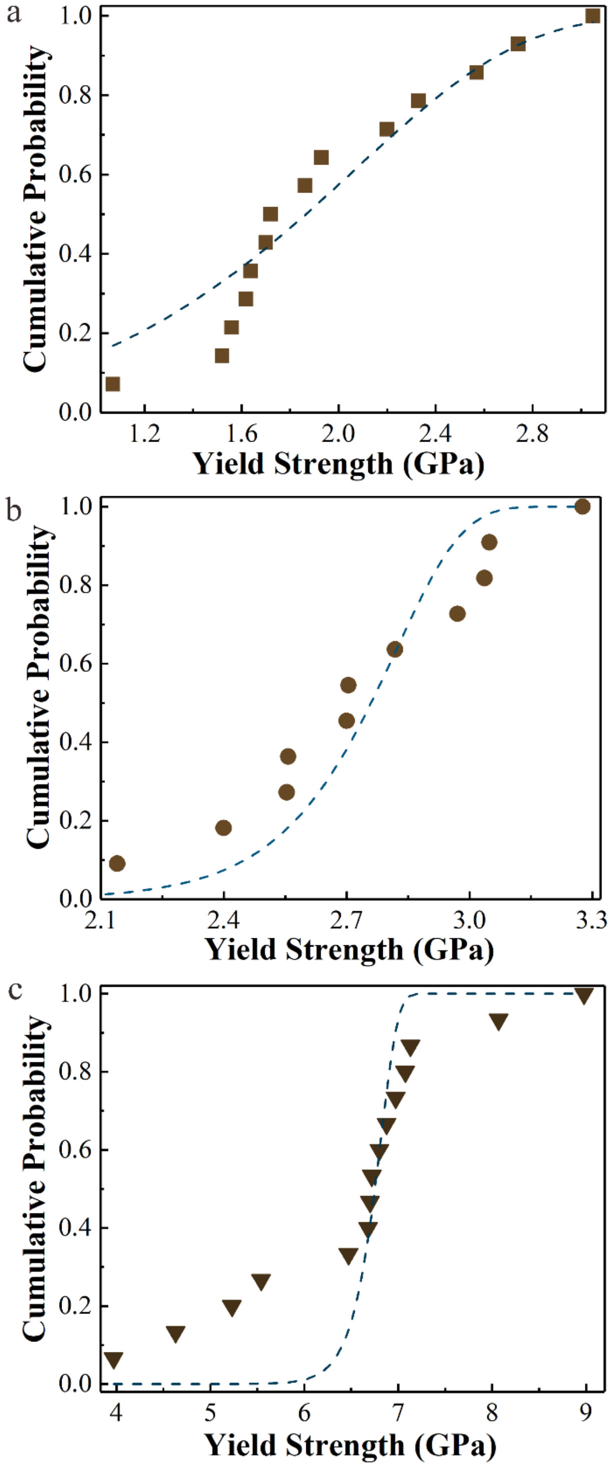

Supplementary Figure 3. Cumulative probability for measured yield strengths of metallic NWs. a, Ag NWs with diameters above 15 nm. **b**, Ag NWs with diameters blow 15 nm. **c**, Pt

NWs. The dotted purple line is a fit for the analytically derived cumulative distribution function $F(\sigma)$.

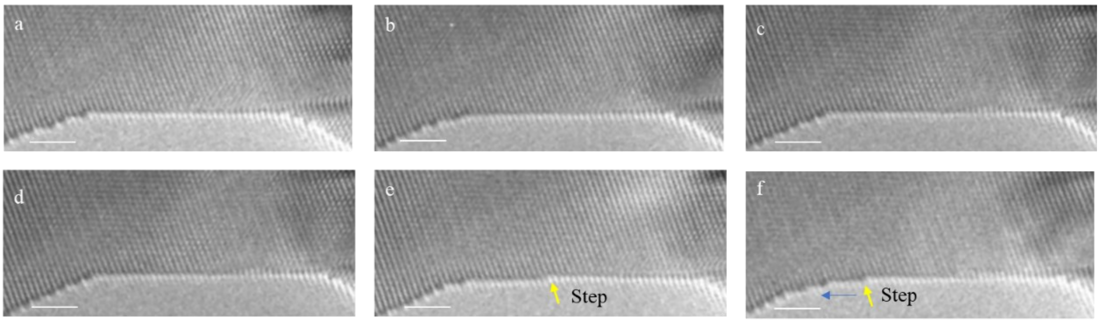

Supplementary Figure 4. The surface dislocation nucleation in the Ag NW with 30 nm. (a-d) The elastic tension of Ag NW before the dislocation nucleation. (e) The formation of one slip-activated surface step. (f) The motion of the slip-activated surface step. The slip-activated surface step was marked by the yellow arrow in e and f. The motion of the step was indicated by the blue arrow. Scale bar 2 nm.

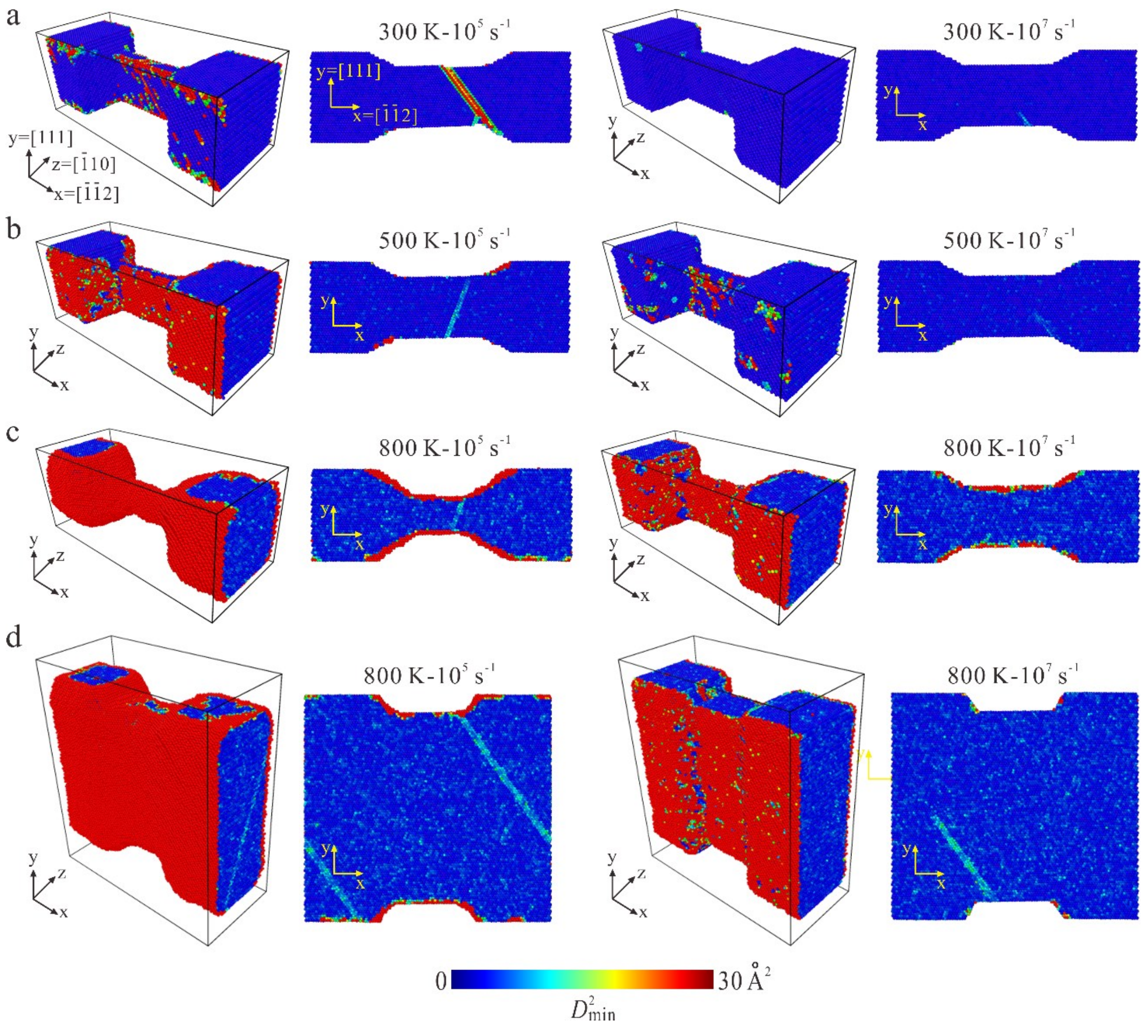

Supplementary Figure 5. Atomic diffusion at the onset of plasticity in Ag NWs under <112> tensile loading at strain rates of 10^5 s^{-1} and 10^7 s^{-1}. **a**,**b**,**c**, 6-nm-diameter Ag NWs at the temperature of 300 K (**a**), 500 K (**b**) and 800 K (**c**). **d**, 20-nm-diameter Ag NWs at the temperature of 800 K. Non-affine squared displacement (D^2_{min}) was used to quantify diffusion in small samples[1]. Each atom in the samples is colored according to its value of D^2_{min}, and the red atoms are diffusive.

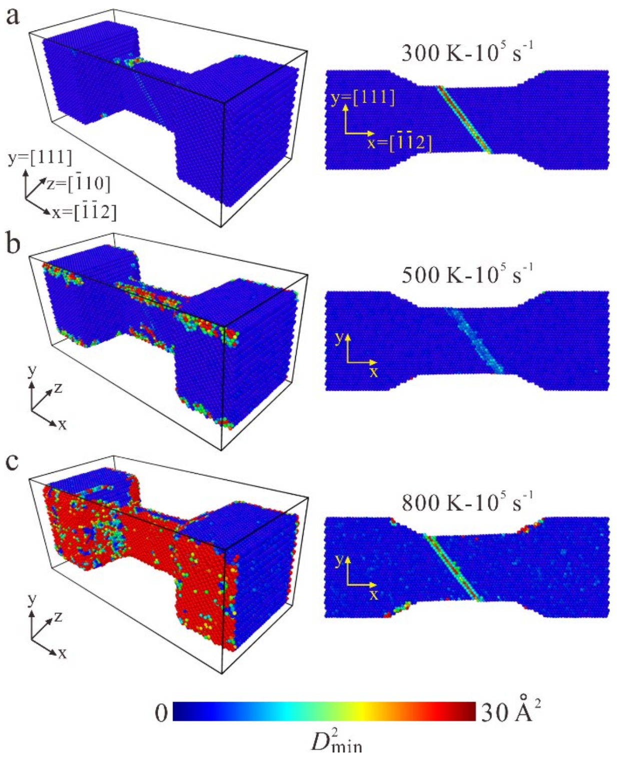

Supplementary Figure 6. Atomic diffusion at the onset of plasticity in 6-nm-diameter Pt NWs deformed along <112> tensile loading under strain rate of 10^5 s^{-1} and at, **a**-**c**, temperature of 300 K (**a**),500 K (**b**) and 800 K (**c**), respectively. Non-affine squared displacement (D^2_{min}) was used to quantify diffusion in small samples[1]. Each atom in the samples is colored according to its value of D^2_{min}, and the red atoms are diffusive.

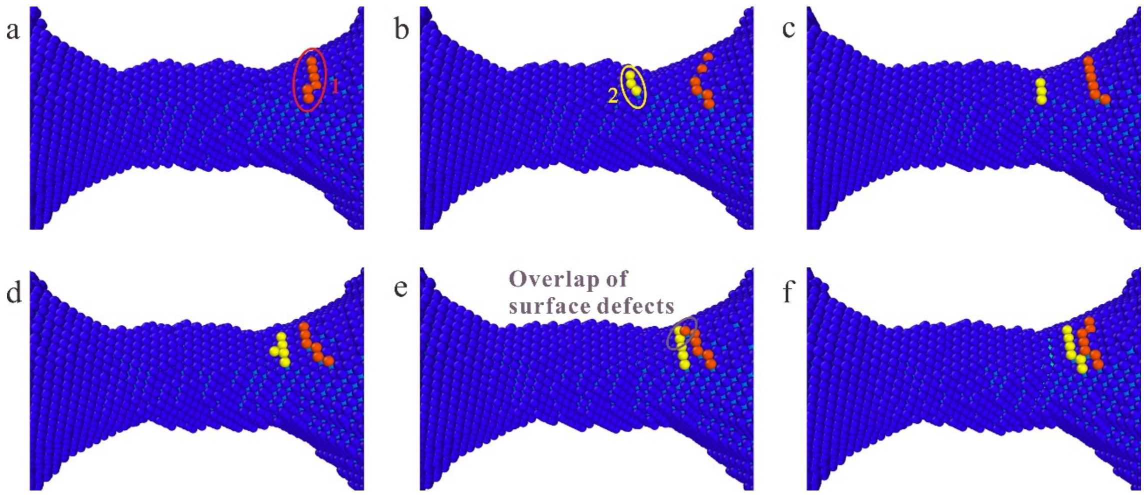

Supplementary Figure 7. Dislocation nucleation assisted by long-range surface atom diffusion. a,**b**, Two surface steps formed by surface diffusion. **c**,**d**, Step 2 migrated to the right side of Ag NW. **e**, Overlap of surface defects (step 1 and step 2). **f**, Dislocation nucleation at the overlapping position of surface defects.

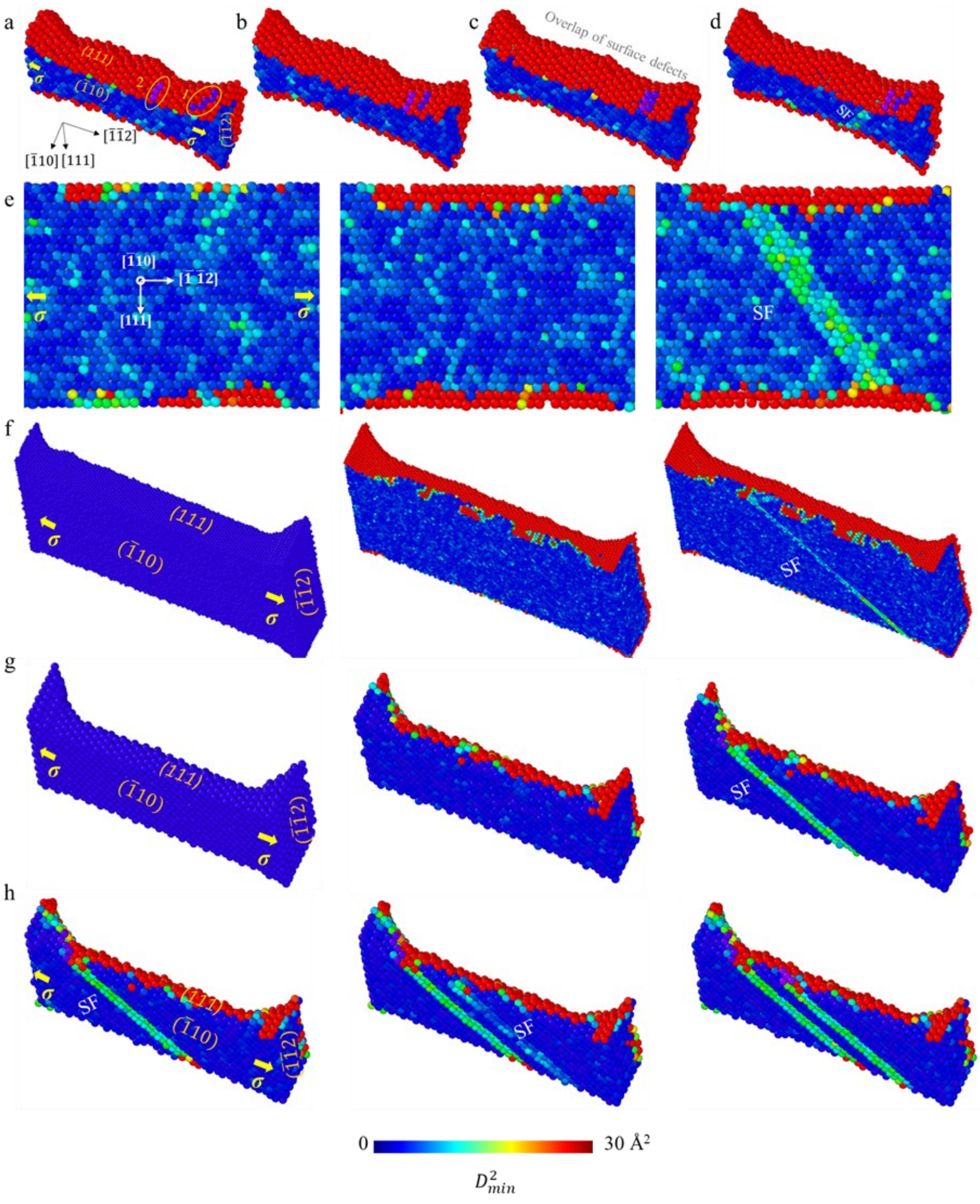

Supplementary Figure 8. Atomic diffusion in metallic NWs deformed along <112> tensile loading at a temperature of 800 K and a strain rate of 10^5 s^{-1}. **a**, Two atomic steps (step 1 and 2) formed by surface diffusion in a 6-nm-diameter Ag NW. **b**, Surface-diffusion-assisted migration of atomic steps (steps 1 and 2) to the right side of NW. **c,** Overlap of surface defects. **d**, Nucleation and propagation of leading partial dislocations at the overlapping position of surface defects. **e**, Dislocation nucleation assisted by single-atom diffusion at the surface of a 6-nm-diameter Ag NW in a random and chaotic way. **f**, Dislocation nucleation in a 20-nm-diameter Ag NW with weak surface diffusion. **g**, Dislocation nucleation in a 6-nm-diameter Pt NW with nearly no surface diffusion. **h**, No surface diffusion during plastic flow in the Pt NW causing that surface steps remained immobile. Surface steps caused by full dislocation activities are colored by dark purple and light purple in **g**,**h**. Non-affine squared displacement (D^2_{min}) was used to quantify diffusion in small samples[1], and the red atoms are diffusive.

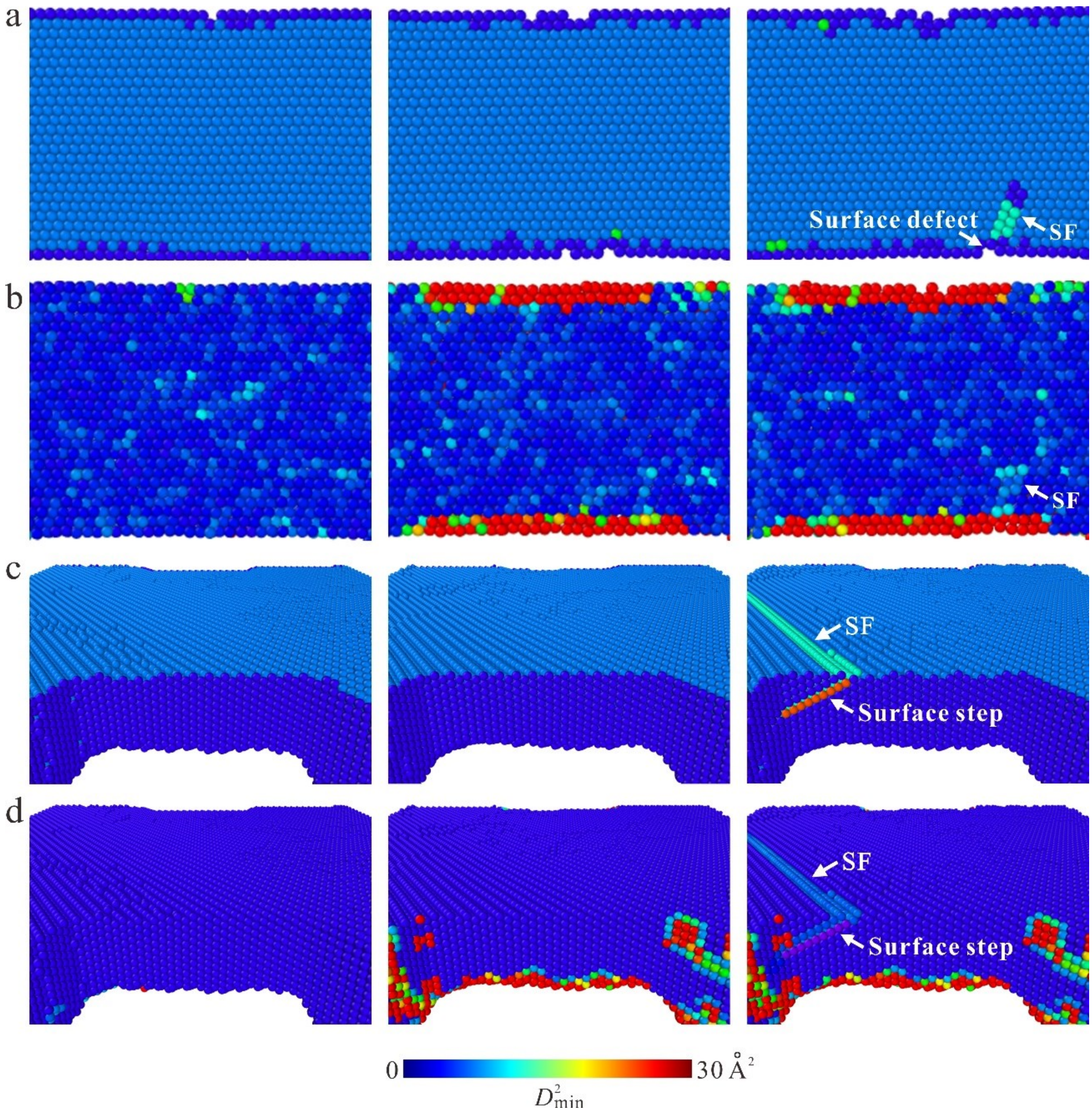

Supplementary Figure 9. MD simulations of dislocation nucleation mechanism in <112>-oriented Ag NWs at 800 K and a strain rate of 10^7 s^{-1}. a, Sequential snapshots (from the left to right) showing dislocation nucleation in a 6-nm-diameter Ag NW, assisted by single-atom diffusion in a random and chaotic way. **b**, Snapshots showing atomic diffusion in the deformed Ag NWs corresponding to **a**. **c**, Sequential snapshots (from the left to right) showing dislocation nucleation in a 20-nm-diameter Ag NW where weak surface diffusion occurred. **d**, Snapshots showing atomic diffusion in the deformed Ag NWs corresponding to **c**. Non-affine squared displacement (D^2_{min}) was used to quantify diffusion in small samples[1]. Each atom in the samples is colored according to its value of D^2_{min}, and the red atoms are diffusive.

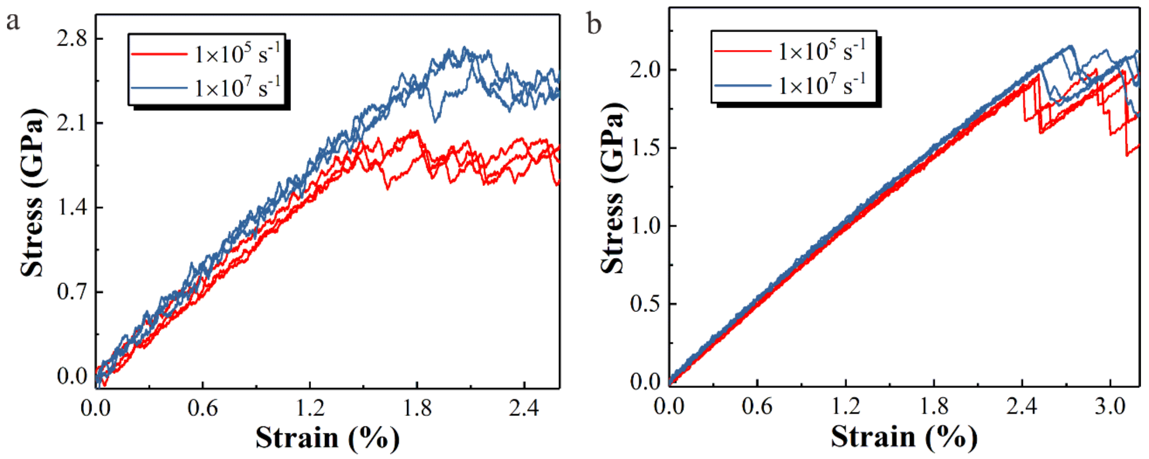

Supplementary Figure 10. Stress-strain curves obtained by MD simulations of tensile straining of Ag NWs at strain rates of 10^5 s^{-1} and 10^7 s^{-1}. a, 6-nm-diameter Ag NWs. **b**, 20-nm-diameter Ag NWs. With the increase in strain rate, the nucleation stress controlled by surface-diffusion-assisted nucleation increased from 1.98 GPa to 2.73 GPa in 6-nm-diameter Ag NW, while the nucleation stress controlled by surface dislocation nucleation increased from 1.97 GPa to 2.15 GPa in 20-nm-diameter Ag NW. Surface-diffusion-assisted nucleation mechanism exhibit a higher strain-rate sensitivity of nucleation stress.

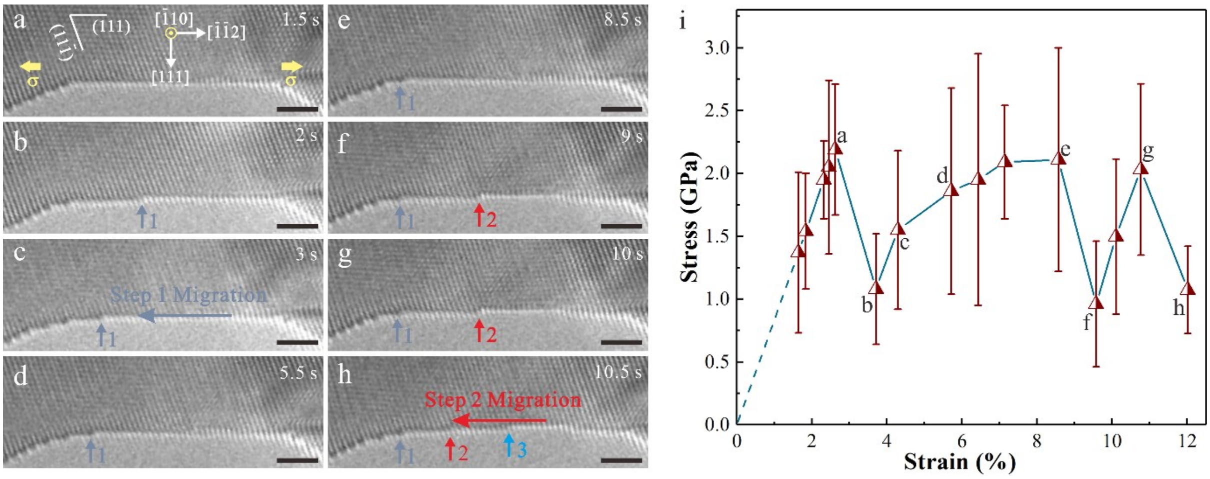

Supplementary Figure 11. Surface atom diffusion induced abnormal softening during plastic flow in an Ag NW. a, Pristine Ag NW with a diameter of 30 nm as viewed along $[\bar{1}10]$ and loaded along $[\bar{1}\bar{1}2]$-orientation at room temperature and a strain rate of ~10^{-3} s^{-1}. **b**, One

surface steps (step 1) formed by a full dislocation activity. **c-e**, Continuous migration of step 1 to the right side of the NW during tensile loading. **f**, A new surface step (step 2) in NW formed by a full dislocation activity. **g**, No obvious surface diffusion during further loading. **h**, A new surface step (step 3) in NW formed by a full dislocation activity. All scale bars are 2 nm. Each surface step is tracked by an arrow of specific color. **i**, Lattice stress versus applied strain curve during tensile loading; points (**a-h**) indicate the states of deformation shown in the TEM images of **a–h**. The initial strains were nominalized by subtracting the plastic strain in previous plastic deformation history. The error bars represent the variations of the measured lattice stresses at different locations of the nanowire.

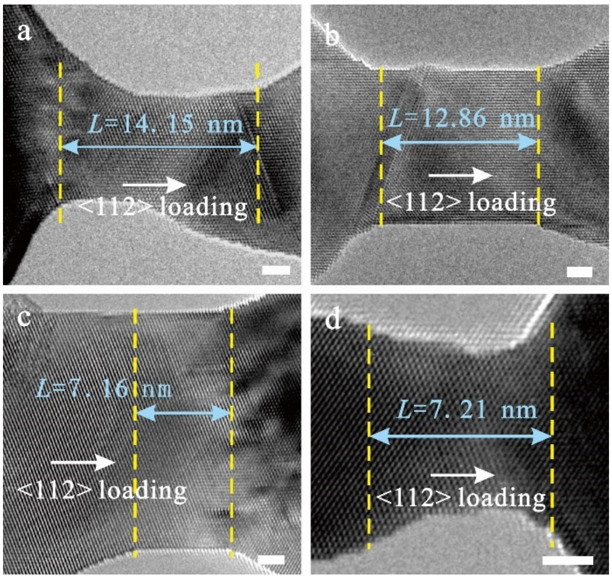

Supplementary Figure 12. The gauge lengths of metallic NWs. a, 6.6-nm-diameter Ag NW. **b**, 13.1-nm-diameter Ag NW. **c**, 30.0-nm-diameter Ag NW. **d**, 5.9-nm-diameter Pt NW. All scale bars are 2 nm.

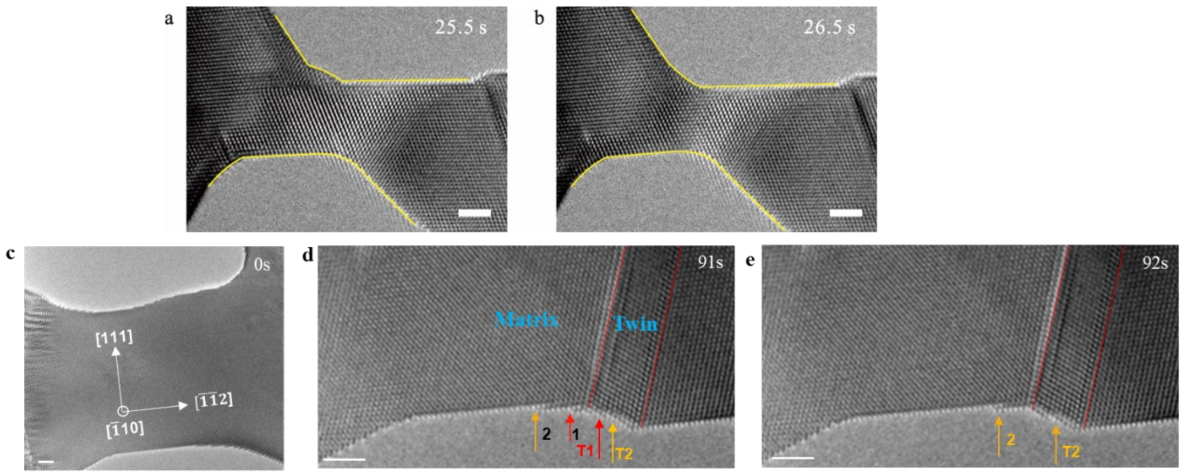

Supplementary Figure 13. The diffusion-contributed plastic strain during tension in Ag NWs. (a-b) Necking behavior in an Ag NW caused by surface atom diffusion under tensile test. (c-e) Non-surface diffusion. Surface steps activated by slipping were indicated by arrows. Steps 1 and T1(T means the twin part) were disappeared near the twin boundary at 92s in e. Scale bar, 2nm

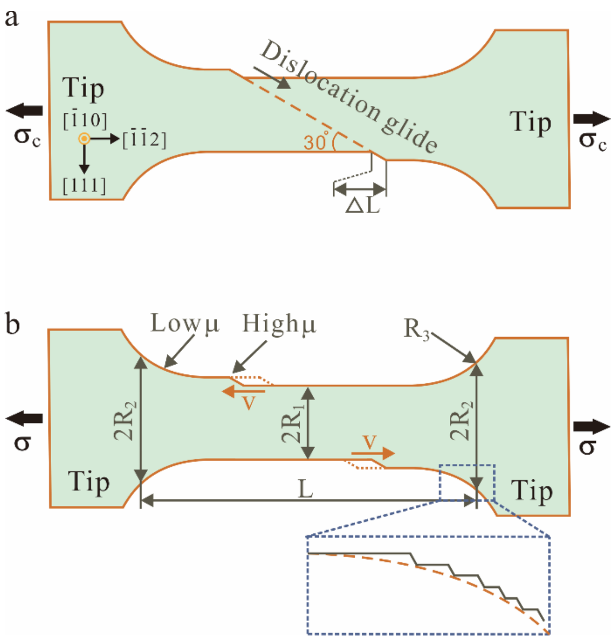

Supplementary Figure 14. Schematics of the plastic deformation process in Ag NWs caused by, a, crystal slip and, b, surface diffusional creep.

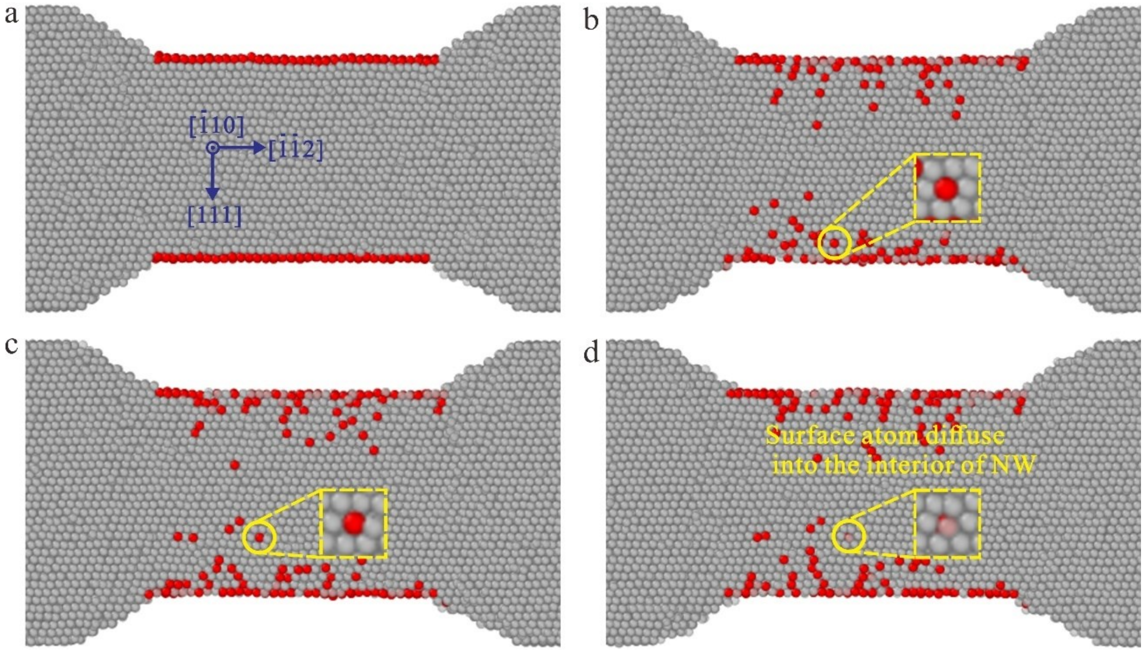

Supplementary Figure 15. Surface diffusional creep in Ag NWs. **a**, Pristine Ag NW with a diameter of 6 nm as viewed along [$\bar{1}$10] and loaded along [$\bar{1}\bar{1}$2]-orientation at temperature of 800 K and a strain rate of 10^5 s^{-1}. The atoms at surface and in the interior of NW is colored by red and grey, respectively. **b,** Some surface atoms diffused onto the ($\bar{1}$10) surface of Ag NW. **c,** The diffused atoms moved on ($\bar{1}$10) surface. **d,** Surface atom diffused into the interior of the NW.

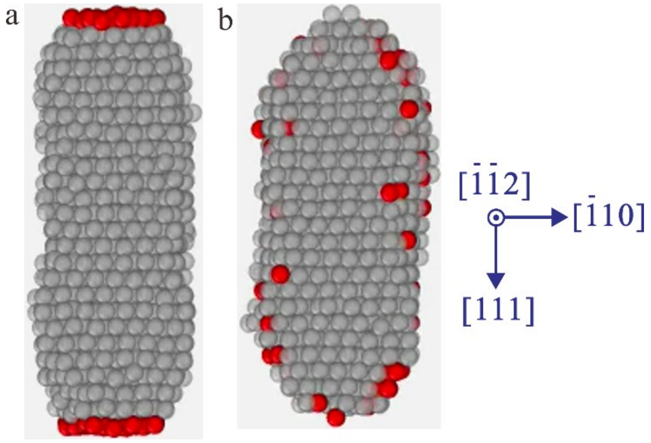

Supplementary Figure 16. Surface diffusional creep in Ag NWs deformed along <112> tensile loading at temperature of 800 K and a strain rate of 10^5 s^{-1}. a, Pristine Ag NW with a diameter of 6 nm as viewed along [$\bar{1}\bar{1}$2]-orientation. The atoms at surface and in the interior

of NW is colored by red and grey, respectively. **b,** Surface atoms diffused into the interior of NW during mechanical loading.

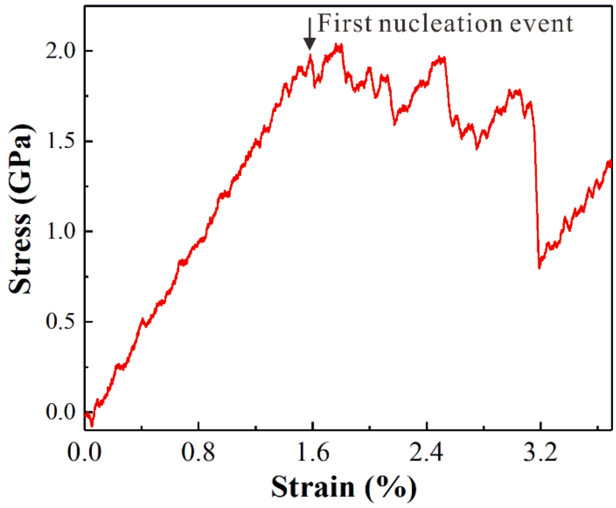

Supplementary Figure 17. Stress-strain curve obtained by the MD simulation of tensile straining of a 6-nm-diameter Ag NW. The arrow indicates the first dislocation nucleation event.

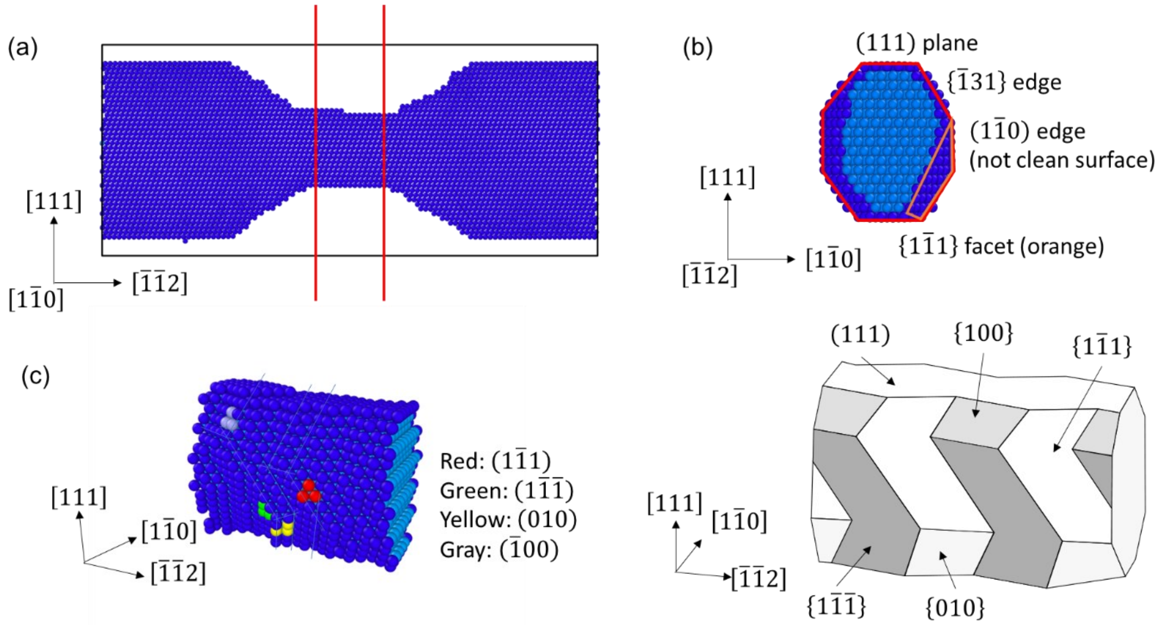

Supplementary Figure 18. The facet evolution of the silver nanowire in MD simulation. (a) The Ag nanowire (6 nm) in MD simulation under tensile loading. (b) Left view for the cutting part marked by the red line in (a). The sample showed different facets. Note that the {100}

plane is not the edge-on plane with [112] axis. (c) The facet of the sample after the further diffusion under loading. Different facets were marked by different color atoms.

Supplementary Tables

Supplementary Table 1. Physical property parameters of Ag and Pt.

	Ag NWs	Pt NWs
Young's modulus E (GPa)	83.61	185.01
Lattice constant a (nm)	0.4079	0.3924
Full dislocation burgers vector b (nm)	0.2921	0.2810
Activation energy barrier for diffusion (eV)	0.064	0.171
Surface mobility μ_s ($m^2 \cdot J^{-1} \cdot s^{-1}$)	$1.4*10^4$	4.3
Surface energy γ (J/m^2)	1.17	2.3

Supplementary Table 2. Activation parameters for dislocation nucleation in metallic NWs at room temperature.

	Ag NWs (<15 nm)	Ag NWs (>15 nm)	Pt NWs
Critical nucleation strength σ_c (GPa)	2.61±0.150	2.96±0.163	5.46±0.135
Athermal nucleation strength σ_{ath} (GPa)	4.27±0.054	3.86±0.085	9.20±0.135
Shear strength along primary slip system τ_{ath} (GPa)	1.68±0.021	1.52±0.033	3.61±0.053
Helmholtz free energy of activation ΔU (eV)	0.16±0.010	0.60±0.022	1.30±0.049
Gibbs free energy of activation ΔG (eV)	0.063±0.005	0.141±0.010	0.316±0.012
Activation volume Ω (b^3)	0.24±0.013	1.00±0.011	1.02±0.003
Nucleation rate prefactor $N\nu_0$ (s^{-1})	3.2±0.080	280.1±32.124	132363.6±2950.494
Nucleation rate ν (s^{-1})	0.28±0.047	1.16±0.510	1.59±0.482

Supplementary Table 3. Experimental and theoretical values of strain increments corresponding to different stress drops caused by dislocation activities.

Sample	6.6-nm-diameter Ag NW	13.1-nm-diameter Ag NW

Stress drop (GPa)	Stress drop 1 (1.56- 0.99)	Stress drop 2 (0.99- 0.88)	Stress drop 3 (1.57- 0.87)	Stress drop 4 (1.46- 1.00)	Stress drop 1 (2.22- 1.01)	Stress drop 2 (2.09- 0.95)	Stress drop 3 (1.97- 1.27)
Dislocation activity	One leading partial	One trailing partial	Two full dislocations	One leading partial	Two full dislocations	Two full dislocations	One full dislocation
Experimental strain increment (%)	0.346	0.027	1.448	0.535	1.201	1.613	0.246
Theoretical strain increment (%)	0.299	0.065	1.705	0.431	1.265	1.349	0.519
Absolute error (%)	0.047	0.018	0.257	0.174	0.064	0.264	0.273
Sample	30.0-nm-diameter Ag NW				5.9-nm-diameter Pt NW		
Stress drop (GPa)	Stress drop 1 (2.19- 1.08)	Stress drop 2 (2.11- 0.96)	Stress drop 3 (2.03- 1.07)		Stress drop 1 (6.36- 3.07)	Stress drop 2 (5.85- 3.21)	Stress drop 3 (5.65- 2.99)
Dislocation activity	One full dislocation	One full dislocation	One full dislocation		One full dislocation	One full dislocation	One full dislocation
Experimental strain increment (%)	1.089	1.004	1.251		0.480	0.757	0.825
Theoretical strain increment (%)	1.036	0.988	1.216		0.444	0.795	0.784
Absolute error (%)	0.053	0.016	0.035		0.036	0.038	0.041

Supplementary Discussion

1. Measurement of the yield strengths and flow stresses in Ag and Pt NWs during mechanical loading

To analyze the lattice strain evolution, lattice spacings were measured in a TEM software of DigitalMicrograph® developed by Gatan Inc (Supplementary Fig. 1). Lattice strain can be determined at atomic scale by analyzing the difference in interplanar spacings over 20 atomic planes between the unstressed state and the deformed state (Supplementary Fig. 1). The lattice stresses (yield strengths and flow stresses) were calculated by multiplying the lattice strains (Supplementary Fig. 2) with the Youngs' modulus of different materials (Supplementary Table 1). To minimize measurement errors, 20-planes measurements were

conducted in NWs. Supplementary Fig. 2 showed the error bars of the measured lattice strain, that came from the variations between repeated measurements. The emission of the first dislocation was regarded as the starting point of the plastic deformation, *i.e.* the yielding point. The lattice strain at a yield point was obtained from the frame prior to first dislocation nucleation, which is different from the yield point obtained using the 0.002 offset criterion[2].Nevertheless, there is no nominal yielding point on the creep curve of materials of which deformation is dominated by diffusion.[3] One basic criterion to determine the elastic instability point under deformation is in terms of the second law of thermodynamics that the change of Helmholtz free energy is larger than or equal to the mechanical work done by the applied stress $\delta F \geq \delta W$.[4] In-situ TEM observation opens up the opportunity to capture the real-time microstructural evolution including the surface diffusion and the dislocation nucleation, providing the simple unified criterion to evaluate the yielding strength of nanocrystals or nanowires as considering the dislocation nucleation.

2. Determining activation parameters for dislocation nucleation in Ag NWs and Pt NWs

According to the transition state theory (TST), the rate of dislocation nucleation is expressed as[5]

$$\nu = N\nu_0 \exp\left(-\frac{\Delta G(\sigma,T)}{k_B T}\right), \quad (1)$$

where N is the number of equivalent nucleation sites ν_0 is the attempt frequency, ΔG (σ, T) is the activation free energy, σ is the applied stress, k_B is the Boltzmann constant, T is the experimental temperature. The activation free energy for dislocation nucleation is given by[6]

$$\Delta G(\sigma, T) = \Delta U(T)\left(1 - \frac{\sigma}{\sigma_{ath}}\right), \quad (2)$$

where $\Delta U(T)$ is the Helmholtz free energy of activation, σ_{ath} is the athermal nucleation strength. The activation free energy (ΔG (σ, T)) can also be expressed as

$$\Delta G\,(\sigma,T)=\Delta U(T) - \Omega\sigma, \tag{3}$$

where Ω is the activation volume defined as the derivative of activation energy with stress ($\Omega=\frac{\Delta U(T)}{\sigma_{\mathrm{ath}}}$)[5]. Combining Eqs. (1) and (3), the rate of dislocation nucleation can be expressed as

$$\nu=N\nu_0\exp\,(-\frac{\Delta U(T)}{\mathrm{k_B}T})\exp\,(\frac{E\dot{\varepsilon}t\Omega}{\mathrm{k_B}T}), \tag{4}$$

in which we assume $\sigma=E\dot{\varepsilon}t$, where E is the Young's modulus of the NWs, $\dot{\varepsilon}$ is the strain rate ($\sim10^{-3}$ in this work) and t is the loading time. Recasting Eq. (4) into the following expression for the cumulative distribution function (CDF)[7]

$$F(t)=1\text{-}\exp(-\int\nu(t)\mathrm{d}t), \tag{5}$$

the cumulative probability of dislocation nucleation related to the stress can be obtained and expressed as

$$F(\sigma)=1\text{-}\exp\{\frac{N\nu_0\mathrm{k_B}T}{E\dot{\varepsilon}\Omega}[\exp\,(-\frac{\Delta U(T)}{\mathrm{k_B}T})\text{-}\exp\,(\frac{E\dot{\varepsilon}t\Omega\text{-}\Delta U(T)}{\mathrm{k_B}T})]\} \tag{6}$$

Fitting Eq. (6) to the experimental CDFs using nonlinear least-squares regression (Supplementary Fig. 3), the activation parameters including Ω, $N\nu_0$, $\Delta U(T)$ and other calculated parameters (ΔG, ν and σ_{ath}) are obtained and summarized in Supplementary Table 2. For Ag NWs (< 15 nm), the fitted value for Ω is 0.24 b^3, where b is the Burgers vector of a full dislocation. This activation volume is on the same magnitude order of the Ω of ~0.1 b^3 for diffusion[8]. Additionally, the fitted activation energy of 0.063 eV is comparable to the activation energy of 0.064 eV for atom self-diffusion on {111} Ag surface hopping along <112> direction[9].

For Ag NWs (>15 nm), the fitted value for Ω is 1 b^3, which is on the same magnitude order of the Ω of 1-10 b^3 for surface dislocation nucleation in NWs without surface diffusion[8, 10, 11]. Besides, the fitted ΔG of 0.141 eV is in the range of 0.16-0.34 eV in the simulations of surface dislocation nucleation in a Cu NW in tension where Ω is about 4 b[35]. Moreover, the ΔG of 0.141 eV in Ag NWs (> 15 nm) is at least an order of magnitude higher than that of 0.063

eV in Ag NWs (< 15 nm) and the ΔG of 0.064 eV for atom self-diffusion. Thus, the activation parameters reported here are effective ones, verifying that dislocation nucleation in Ag NWs (> 15 nm) is not associated with surface diffusion. Likewise, the fitted activation parameters for Pt NWs (ΔG is 0.316 eV and Ω is 1.02 b^3) also demonstrate that surface dislocation nucleation occurred in Pt NWs.

The reason why the fitted $N\nu_0$ in this work (Supplementary Table 2) are much lower than those in MD simulations (10^{11} s^{-1} -10^{13} s^{-1}) may be attributed to the following two reasons. First, the vibrational frequency of surface atoms are different from their bulk counterparts[12]. Second, the individual atomic vibration frequency may not be a suitable attempt frequency given that dislocation nucleation is associated with a collective group of atoms[6]. The calculated ν in Ag NWs (< 15 nm), Ag NWs (> 15 nm) and Pt NWs are 0.28, 1.16 and 1.59 respectively, which are comparable to the short timescale of ~1 s for yielding observed in our experiments. For constant strain rate experiments and simulations, the nucleation rate is on the order of the applied strain rate, which is about 10^7 s^{-1}-10^8 s^{-1} in MD simulations and 10^{-2} s^{-1}-10^{-3} s^{-1} in experiments[11]. Thus, the calculated nucleation rates through mathematical analysis, that are much lower than those in MD simulations (2.5×10^7 s^{-1} -2.5×10^9 s^{-1}), are effective ones[13].

By obtaining the fitted Ω and $\Delta U(T)$, it is also possible to get the athermal strength (σ_{ath}) for Ag NWs and Pt NWs based on the equation $\Omega = \frac{\Delta U(T)}{\sigma_{\text{ath}}}$. The shear strengths, σ_{ath} resolved along with the primary slip system, are 1.68 GPa for Ag NWs (< 15 nm), 1.52 GPa for Ag NWs (> 15 nm) and 3.6 GPa for Pt NWs respectively. These values of 1.6 GPa and 1.52 GPa for heterogeneous nucleation in Ag NW closely approach the reported ideal shear strength of 1.65 GPa in Ag from ab initio calculations[14]. Likewise, the shear strength of 3.61 GPa in Pt NW for dislocation nucleation is comparable to the reported value of 2.75 GPa in Pt[15].

3. Contribution of full/partial dislocation activities to the plastic strain during tensile tests

As is well known, a metallic NW experiences both elastic deformation and plastic deformation during mechanical loading. If a metallic NW experiences elastic deformation, the strain during tensile loading is given by $\varepsilon_{\text{elastic}}=\frac{\sigma_2-\sigma_1}{E}$, where σ_1 and σ_2 are stresses before and after deformation respectively and E is the Young's modulus of metallic NW. As the lattice stress inside a NW is high enough to reach a yield point, the nucleation, propagation andannihilation of a full/partial dislocation in NW can result in permanent plastic deformation[16]. The plastic strain induced by a dislocation activity in a $[11\bar{2}]$-orientated metal NW is quantitatively calculated as follows. One of the activated full dislocations with the largest Schmid factor on $(11\bar{1})$ is $\frac{1}{2}[101]\,a$, where a is the lattice constant of the metal. Each full dislocation activity can contribute to an axial elongation of $\frac{\sqrt{6}}{6}a$ along the $[11\bar{2}]$ loading direction (Supplementary Fig. 12a). Thus, each full dislocation activity contributes to a plastic strain of $\frac{\sqrt{6}}{6}\frac{a}{L}$, where L is the gauge length. Likewise, the Burgers vectors of the activated leading and trailing partial dislocations on $(11\bar{1})$ are $\frac{1}{6}[2\bar{1}1]\,a$ and $\frac{1}{6}[\bar{1}21]\,a$, respectively based on Schmid law. Each leading and trailing partial activity can contribute to an axial elongation of $\frac{5\sqrt{6}}{36}a$ and $\frac{\sqrt{6}}{36}a$, respectively along the $[11\bar{2}]$ loading direction (Supplementary Fig. 14a). Accordingly, each leading and trailing partial dislocation activity contribute to a plastic strain of $\frac{5\sqrt{6}}{36}\frac{a}{L}$ and $\frac{\sqrt{6}}{36}\frac{a}{L}$, respectively. Previous MD studies reported that yielding resulted in precipitous stress drop under the displacement controlled loading[17]. Compared with the recording interval of 4 ps in MD simulations[17], the frame rate of 0.5s/frame in the CCD camera was much larger. The accurate lattice stresses before and after a yield point were not captured in our work such that the significant stress drops with nearly no strain increments after yielding were not observed in the stress-strain curves in our work (Fig. 2m, Fig. 3g, Fig. 5m and Supplementary Fig. 11i). The strain increments corresponding to stress drops in the present

study were composed of the elastic strain induced by elastic deformation and the plastic strain caused by dislocation activities, which can be expressed as

$$\varepsilon=\frac{\sigma_2\text{-}\sigma_1}{E}+\varepsilon_{\text{plastic}} \tag{7}$$

where σ_1 and σ_2 are the lattice stress in a NW before and after a plastic event, respectively and $\varepsilon_{\text{plastic}}$ is the plastic strain caused by full/partial dislocation slip activities. The experimentally measured and theoretical strain increments corresponding to different stress drops were tableted in Supplementary Table 3. The average absolute errors between the theoretical values and the experimental values of the strain increments is less than 0.1% (Supplementary Table 3), verifying that the evaluation method for the stress-strain curves in this work is feasible and reliable.

4. The details of the mechanical behaviors in Ag NWs with sample diameters of 6.6 nm, 13.1 nm and 30.0 nm

Upon tensile loading of a 6.6-nm-diameter Ag NW, steps 2 and 3 migrated to the right side of the NW, while step 1 migrated to the left side (Fig. 2a-d). When step 3 migrated to the initial location of step 2 (marked by the green circle), a leading partial dislocation was nucleated from this site leaving behind a surface step with a height of one-third of an atomic-layer (Fig. 2g), which was subsequently eliminated by a trailing partial dislocation at the same site to form a full dislocation leaving behind a one-atomic-layer-step denoted by step 4 (Fig. 2h). With further tensile loading, the migration of surface steps was not observed, as shown in Fig. 2i. When the accumulated lattice strain in the Ag NW was high enough again, two full dislocations nucleated and propagated through the Ag NW, leaving behind two one-atomic-layer-steps denoted by step 5 and 6 respectively (Fig. 2j). The migration of surface steps was not observed during the further loading (Fig. 2k), and then one partial dislocation nucleated and propagated

through the Ag NW leaving behind a stacking fault and a surface step with one-third of the height of an atomic-layer (Fig. 2l).

A 13.1-nm-diameter Ag NW was chosen to investigate the influence of surface atom diffusion on plastic flow as shown in Fig. 5. The Ag NW only experienced elastic deformation without detectable surface diffusion before yielding, as shown in Fig. 5a. With subsequent tensile straining, two dislocations nucleated and propagated in Ag NW leaving behind two one-atomic-layer-steps denoted by steps 1 and 2 respectively (Fig. 5b), and then these two steps migrated to the right side of the NW (Fig. 5c-h). When the accumulated elastic strain in Ag NW was high enough, full dislocation activities occurred, leaving behind two one-atomic-layer-steps (denoted by steps 3 and 4 respectively) at the surface of the Ag NW (Fig. 5i). The steps 1, 2, 3 and 4 further migrated to the right side of the Ag NW (Fig. 5i-k) before the next plastic event (Fig. 5l). Eventually, one-atomic-layer-step (step 5) formed by a full dislocation activity emerged at the surface.

The mechanical behaviors of the 30-nm-diameter Ag NW were investigated, as shown in Supplementary Fig. 10. A one-atomic-layer-step (denoted by step 1) appeared at the surface of Ag NW after yielding, (Supplementary Fig. 10b) before which the Ag NW suffered from elastic deformation (Supplementary Fig. 11a). With the increase in applied strain, step 1 migrated to the right side of the sample (Supplementary Fig. 11c-e). Subsequently, a one-atomic-layer-step formed at the surface of the Ag NW caused by a full dislocation activity (Supplementary Fig. 11f). Upon further deformation, dislocation nucleated in Ag NW (Supplementary Fig. 11h), before which the migration of surface step was not observed (Supplementary Fig. 11g).

5. Surface-diffusion-assisted dislocation nucleation

(1) The driving force for surface diffusion

The potential energy change for a surface atom migrating from the middle to either end of the NW can be expressed by[18, 19]

$$\Delta E=\frac{\gamma}{\rho}\left(\frac{1}{R_2}-\frac{1}{R_1}-\frac{1}{R_3}\right)+\frac{\sigma^2}{2\rho E}\left(\frac{R_1^4}{R_2^4}-1\right), \quad (8)$$

where R_1 is the radius of curvature in the middle of the NW, R_2 is the radius of curvature at both ends, R_3 is the radius of curvature of a stable curved contour where a series of surface steps connects the NW to the tips (Supplementary Fig. 14b), ρ is the bulk density, γ is the surface energy, σ is the applied stress during tensile test and E is Young's modulus of the metallic NW. To obtain the relation of the driving force with the sample size, we set the following assumptions: The NW's diameter is $D = 2R_1$, $R_2 = 2R_1$, and $R_3 = 2R_1$. The ΔE can be simply expressed by

$$\Delta E=-\frac{2\gamma}{\rho D}-\frac{15\sigma^2}{32\rho E}, \quad (9)$$

where the driving force of diffusion would increase with decreasing the sample size of NWs.

(2) The size dependence of the diffusivity

The relation of the melting point of metal particles with the size can be expressed by[20, 21]

$$\frac{T_m(D)}{T_m(\infty)} = 1 - \frac{4}{\rho_s L}\left\{\gamma_s - \gamma_l\left(\frac{\rho_s}{\rho_l}\right)^{2/3}\right\}\frac{1}{D}, \quad (10)$$

where $T_m(\infty)$ is the bulk melting temperature, $T_m(r)$ is the melting temperature of the particle with a diameter D, L is the molar heat of fusion, ρ_s, ρ_l are the solid and liquid densities. Then, the relation of diffusivity with the melting temperature can be expressed by[20]

$$D_D = D_m exp\left[-\Delta H(r)\left(\frac{1}{T}-\frac{1}{T_m(D)}\right)\right], \quad (11)$$

where D_m is the diffusion coefficient at the bulk melting temperature, D_D is the diffusion coefficient of the particle with a diameter D, ΔH is the activation enthalpy of diffusion, T is the temperature and D_0 is the prefactor. Based on equations (10) and (11), the surface diffusivity is inversely proportional to the sample size.

The athermal nucleation stress with the surface step $\hat{\sigma}_{\text{ath}}$ can be expressed by

$$\hat{\sigma}_{ath} = (1 - \Delta \cdot \rho_D) \cdot \sigma_{ath}, \tag{12}$$

where Δ is the stress decrement due to the step,[22] and ρ_D is the probability function of the involvement of the step for the nucleation which is proportional to the surface diffusivity.

The nucleation site number with the diffusion activities can be expressed by

$$\widetilde{N} = N + \frac{D_D \cdot t}{\pi \cdot D \cdot L}, \tag{13}$$

where N is the nucleation site number without diffusion, t is the unit time.

Supplementary References

1. Guo W, Wang Z, Li J. Diffusive versus displacive contact plasticity of nanoscale asperities: temperature-and velocity-dependent strongest size. *Nano letters* **15**, 6582-6585 (2015).

2. Dieter GE, Bacon DJ. *Mechanical metallurgy*. McGraw-hill New York (1986).

3. Ashby MF. A first report on deformation-mechanism maps. *Acta Metallurgica* **20**, 887-897 (1972).

4. Hill R, Milstein F. Principles of stability analysis of ideal crystals. *Physical Review B* **15**, 3087 (1977).

5. Zhu T, Li J, Samanta A, Leach A, Gall K. Temperature and strain-rate dependence of surface dislocation nucleation. *Physical Review Letters* **100**, 025502 (2008).

6. Chen LY, He M-r, Shin J, Richter G, Gianola DS. Measuring surface dislocation nucleation in defect-scarce nanostructures. *Nature materials* **14**, 707 (2015).

7. Mason J, Lund A, Schuh C. Determining the activation energy and volume for the onset of plasticity during nanoindentation. *Phys Rev B* **73**, 054102 (2006).

8. Zhu T, Li J. Ultra-strength materials. *Progress in Materials Science* **55**, 710-757 (2010).

9. Kim SY, Lee I-H, Jun S. Transition-pathway models of atomic diffusion on fcc metal surfaces. I. Flat surfaces. *Physical Review B* **76**, 245407 (2007).

10. Weinberger CR, Jennings AT, Kang K, Greer JR. Atomistic simulations and continuum modeling of dislocation nucleation and strength in gold nanowires. *Journal of the Mechanics and Physics of Solids* **60**, 84-103 (2012).

11. Weinberger CR, Cai W. Plasticity of metal nanowires. *Journal of Materials Chemistry* **22**, 3277-3292 (2012).

12. Rabkin E, Srolovitz DJ. Onset of plasticity in gold nanopillar compression. *Nano letters* **7**, 101-107 (2007).

13. Li QJ, Xu B, Hara S, Li J, Ma E. Sample-size-dependent surface dislocation nucleation in nanoscale crystals. *Acta Mater* **145**, 19-29 (2018).

14. Ogata S, Li J, Hirosaki N, Shibutani Y, Yip S. Ideal shear strain of metals and ceramics. *Physical Review B* **70**, 104104 (2004).

15. Černý M, Pokluda J. The theoretical tensile strength of fcc crystals predicted from shear strength calculations. *Journal of Physics: Condensed Matter* **21**, 145406 (2009).

16. Cao G*, et al.* Superplasticity in gold nanowires through the operation of multiple slip systems. *Advanced Functional Materials* **28**, 1805258 (2018).

17. Cao A, Wei Y, Mao SX. Alternating starvation of dislocations during plastic yielding in metallic nanowires. *Scripta Materialia* **59**, 219-222 (2008).

18. Torres J*, et al.* The puzzling stability of monatomic gold wires. *Surf Sci* **426**, L441-L446 (1999).

19. Zhong L, Sansoz F, He Y, Wang C, Zhang Z, Mao SX. Slip-activated surface creep with room-temperature super-elongation in metallic nanocrystals. *Nat Mater* **16**, 439-445 (2017).

20. Dick K, Dhanasekaran T, Zhang Z, Meisel D. Size-dependent melting of silica-encapsulated gold nanoparticles. *Journal of the American Chemical Society* **124**, 2312-2317 (2002).

21. Buffat P, Borel JP. Size effect on the melting temperature of gold particles. *Physical review A* **13**, 2287 (1976).

22. Li C, Xu G. Critical conditions for dislocation nucleation at surface steps. *Philosophical Magazine* **86**, 2957-2970 (2006).
