# Supplementary material for: Atomistic processes of surface-diffusion-induced abnormal softening in nanoscale metallic crystals
Source: Nat Commun. 2021 Sep 2;12:5237. doi: 10.1038/s41467-021-25542-2 (PMC8413310; doi:10.1038/s41467-021-25542-2)
Supplement: Supplementary file 2 — Description of Additional Supplementary Files [file 41467_2021_25542_MOESM2_ESM.pdf]

Description of Additional Supplementary Files

File Name: Supplementary Movie 1
Description: Dislocation nucleation assisted by surface diffusion in a -oriented 6.6-nm-diameter Ag NW at room temperature and a strain rate of ~10-3 s-1.

File Name: Supplementary Movie 2
Description: Pure displacive deformation in a -oriented 5.9-nm-diameter Pt NW at room temperature and a strain rate of ~10-3 s1.

File Name: Supplementary Movie 3
Description: Molecular dynamics simulation of tensile straining of 6-nm-diameter Ag NW at 800 K and a strain rate of 105 s-1 showing dislocation nucleation assisted by long-range surface diffusion.

File Name: Supplementary Movie 4
Description: Molecular dynamics simulation of tensile straining of 6-nm-diameter Ag NW at 800 K and a strain rate of 105 s-1 showing dislocation nucleation assisted by localized diffusion of individual atoms at free surface in a random and chaotic way.

File Name: Supplementary Movie 5
Description: Molecular dynamics simulation of tensile straining of 20-nm-diameter Ag NW at 800 K and a strain rate of 105 s-1 showing dislocation nucleation with weak surface diffusion.

File Name: Supplementary Movie 6
Description: Molecular dynamics simulation of tensile straining of 6-nm-diameter Pt NW at 800 K and a strain rate of 105 s-1 showing surface dislocation nucleation and plastic flow with nearly no diffusional events.

File Name: Supplementary Movie 7
Description: Surface atom diffusion induced abnormal softening during plastic flow in a -oriented 13.1-nm-diameter Ag NW at room temperature and a strain rate of ~10-3 s-1. The movie is played at a speed of 3X.

File Name: Supplementary Movie 8
Description: Surface atom diffusion induced abnormal softening during plastic flow in a -oriented 30-nm-diameter Ag NW at a strain rate of ~10-3 s-1.
